# Mobile-CRISPRi as a tool for genetic manipulation in the intracellular pathogen *Piscirickettsia salmonis*

**DOI:** 10.1128/aem.01560-25

**Published:** 2025-12-22

**Authors:** Javiera Ortiz-Severin, Paulette Geoffroy, Pamela Aravena, Christian Hodar, Daniel E. Palma, Mauricio González, Verónica Cambiazo

**Affiliations:** 1 Laboratorio de Bioinformática y Expresión Génica, Instituto de Nutrición y Tecnología de los Alimentos (INTA), Universidad de Chile 14655, Santiago, Chile; 2 Laboratorio de Bioinformática y Bioestadística Genómica, INTA, Universidad de Chile 14655, Santiago, Chile; 3 Millennium Institute Center for Genome Regulation, Santiago, Chile; Indiana University Bloomington, Bloomington, Indiana, USA

**Keywords:** Mobile-CRISPRi, fish pathogen, Ferric uptake regulator, iron homeostasis genes

## Abstract

*Piscirickettsia salmonis* is the causative agent of salmonid rickettsial septicemia (SRS), the main bacterial disease affecting the salmon industry in Chile. In this work, we implemented a Mobile-CRISPRi system to generate gene silencing using a catalytically inactive dCas9 protein and an isopropyl β-D-1-thiogalactopyranoside (IPTG)-inducible single-guide RNA (sgRNA). We demonstrate the efficacy of the CRISPRi system in *P. salmonis* by silencing an exogenous reporter (sfGFP) and an endogenous regulator (Fur) that controls intracellular iron homeostasis in bacteria. The inducible expression of *dCas9* and the *sfGFP*-directed sgRNA caused a 98.7% decrease in fluorescence in the knockdown strain. This silencing system was effective in seven *P. salmonis* strains from both genogroups. Furthermore, the same system was used to construct *fur* knockdown strains. A 50-fold decrease in *fur* expression level was determined in these strains when the expression of the *fur* gRNA was induced with IPTG. By RNA-seq, we detected a significant increase in the expression of genes encoding the Fe^2+^ and Fe^3+^ acquisition systems and iron mobilization in the *fur1* knockdown after IPTG induction. All the genes with over 2-fold increased expression in the RNA-seq presented the Fur box consensus sequence in their regulatory region. The implementation of the Mobile-CRISPRi system in *P. salmonis* has been demonstrated to be effective, thus providing a tool with potential application for the analysis of gene function in this pathogen. It is anticipated that these analyses will be valuable in identifying genes involved in the mechanisms of pathogenesis of *P. salmonis*.

**IMPORTANCE:**

Salmonid rickettsial septicemia (SRS) is an infectious disease caused by the marine bacterium *Piscirickettsia salmonis*. This Gamma-proteobacteria is a fastidious and facultative intracellular pathogen that has a nearly worldwide distribution, particularly impacting Chilean salmonid aquaculture. Its fastidious nature has made it hard to grow in labs, hindering research into its virulence and treatment, especially because of the lack of molecular techniques to study gene function. We show here the successful implementation of the Mobile-CRISPRi system for gene silencing. Significantly, we have adapted this technique for use with the marine pathogen *P. salmonis*, inserting exogenous genes into the bacterium's chromosome to ensure their constitutive and inducible expression and silencing both exogenous and endogenous gene expression. The Mobile-CRISPRi system was also used to study the iron regulator Fur, confirming Fur's relevance to the iron metabolism in the pathogen.

## INTRODUCTION

The CRISPR-Cas system has been broadly utilized in a variety of species as a genome-engineering tool (1). A related method, CRISPR interference (CRISPRi), uses an inactive Cas9 protein (dCas9) that has lost nuclease activity. Once the single-guide RNA (sgRNA) and dCas9 complex is formed, it represses transcription by inhibiting the initiation or progression of RNA polymerase at a specific target locus. This system yields loss-of-function phenotypes that are suitable for a systematic genetic analysis aiming to uncover gene phenotypes (2–4). More recently, an additional system named Mobile-CRISPRi has been established, including a collection of modular vectors that enable gene knockdowns in diverse gram-negative bacteria by integrating isopropyl β-D-1-thiogalactopyranoside (IPTG)-inducible *dcas9* gene and sgRNA sequences into the genome using the Tn7 transposon system (4). Therefore, Mobile-CRISPRi has the advantage of facilitating the analysis of gene function in non-model species and strains (5). Although the CRISPRi technology has been primarily characterized in the model bacteria *Escherichia coli* and *Bacillus subtilis*, there has been a recent proliferation of studies that have demonstrated the extension of these tools to non-model bacteria, including those from the genera Mycobacterium and Streptomyces, the phyla Actinomycetota, Cyanobacteriota, Bacillota, and a wide variety of Pseudomonadota (6).

The objective of this study was to establish a stable and effective Mobile-CRISPRi-induced knockdown for *Piscirickettsia salmonis*, a fastidious facultative intracellular bacterium that is the causal agent of salmonid rickettsial septicemia (SRS), the main bacterial disease affecting the salmon industry in Chile (7). *P. salmonis* has a nearly global distribution, being isolated in other important salmon-farming countries (8–10), where it is regarded as a potential emerging pathogen (11, 12). It is acknowledged that the range of molecular tools available to regulate gene expression in *P. salmonis* is limited and that there is a clear constraint on our ability to interrogate the function of essential and conditionally essential genes.

In this study, using a Mobile-CRISPRi variant containing a constitutively expressed *sfGFP* reporter and an sgRNA targeting *sfGFP* (4), we tested the Mobile-CRISPRi system in different strains of *P. salmonis*, encompassing the two genogroups, LF89-like and EM90-like, previously described for this bacterium (13). Afterward, we used this system to study the function of the Ferric uptake regulator (Fur) in *P. salmonis*, which has been proven in other bacteria to reversibly bind Fe^2+^ and repress the expression of iron acquisition genes, allowing their expression only when the level of free intracellular iron is low (14). Fur has also been implicated in the regulation of virulence genes, stress resistance, toxin production, and biofilm formation (15), and throughout small RNAs such as *rhyB*, or *IsrR*, Fur has been shown to indirectly regulate processes such as the TCA cycle and the response to oxidative stress (16–19). Due to its importance in virulence, the Fur regulon has been studied in several model bacterial species (20–24). Iron metabolism is especially important in intracellular pathogens, such as *P. salmonis*, because iron acquisition from the host is essential for intracellular growth and survival, and thus, iron starvation becomes a virulence signal in these pathogens (25, 26). In a previous study, we predicted a set of iron acquisition genes in *P. salmonis* whose increased expression under iron-starved conditions, and the presence of putative Fur boxes upstream, suggested that most of these genes were part of the Fur regulon (27). Moreover, during *P. salmonis* infection in a macrophage-like cell line, metal acquisition proteins were one of the most highly expressed families of virulence factors (28). In this study, we employed a combination of CRISPRi-based knockdown of *fur* and RNA sequencing to elucidate the effects of *fur* knockdown on global gene expression. In conclusion, the present study demonstrates the efficacy of Mobile-CRISPRi in *P. salmonis*, thus validating the applicability of this system for gene functional analysis.

## MATERIALS AND METHODS

### Bacterial strains and growth conditions

Oligonucleotides used in this study for PCR, RT-qPCR, and cloning can be found in Table S1. All bacterial strains and plasmids are listed in Tables S2 and S3, respectively. *P. salmonis* strains were routinely grown at 18°C with agitation, on Nutrient *Piscirickettsia* Broth (NPB; 30 g/L Tryptic Soy Broth, 256.6 mM NaCl, 8.25 mM L-cysteine, 37 µM FeCl_3_, and 2.5% of inactivated fetal bovine serum [FBS]). Bacteria were recovered from Nutrient *Piscirickettsia* Agar plates (NPA, NPB supplemented with 5% agar) and used to inoculate NPB. Cultures were incubated in a shaking incubator at 180 rpm and 18°C for 36 h (exponentially growing bacteria) or 96 h (stationary state bacteria) before harvesting. Purity of *P. salmonis* cultures was tested using a PCR-RFLP assay, as described in Mandakovic *et al*. (29). *E. coli* strains were routinely grown in Luria-Bertani (LB) broth at 37°C with shaking. When required, the appropriate media were supplemented with gentamicin (30 μg/mL), ampicillin (100 μg/mL), or IPTG at 100 μM unless otherwise noted.

### Transformation of *E. coli* SM10

The procedures for purifying pJMP2754, pJMP2774, and pTNS3 plasmid DNA, as well as for transforming *E. coli* SM10, are described in the supplemental methods.

### Plasmid conjugation into *P. salmonis*

The Mobile-CRISPRi system was transferred to *P. salmonis* by tri-parental conjugation using the following *E. coli* donor strains: (i) SM10 strain containing the plasmid of interest (pJMP2754, pJMP2774, pJMP2782:*fur1*, or pJMP2782:*fur2*) and (ii) SM10 strain containing pTNS3 encoding the Tn7 transposase. Before plasmid conjugation, a single colony of each *E. coli* strain was inoculated in 3 mL of LB broth with the appropriate antibiotic and grown overnight (ON) at 37°C. The ON cultures were diluted to an optical density at 600 nm (OD_600_) of 0.01 in 5 mL of NPB medium supplemented with the appropriate antibiotic and grown at 20°C with shaking at 180 rpm for 3 days. *P. salmonis* recipient strain was also grown to the stationary phase in NBP at 20°C with shaking for 4 days. One milliliter of donor strain culture was centrifuged at 6,000 × *g* for 3 min at 4°C, washed three times with NPB, resuspended in 1 mL of NPB, and mixed with the recipient strain in a ratio of 1:1:1 to a final volume of 300 µL. The mix was applied on a 0.45 µm S-Pak membrane filter (Merck) on NPA plates and incubated at 18°C for 40 h. At the end of the incubation period, the filter was placed in a 50 mL conical tube containing 10 mL NPB and vortexed before 200 µL aliquots were poured on NPA plates supplemented with gentamicin (30 µg/mL) and trimethoprim (25 µg/mL). Plates were incubated at 18°C for 2–3 weeks before transconjugants were harvested. The matings were carried out in triplicate, using three independent cultures of each donor and recipient strain. At least 10 colonies per conjugation event were picked up and inoculated on a 96-well plate with 200 µL NPB supplemented with gentamicin and trimethoprim and grown for 4 days at 20°C with shaking at 180 rpm. Grown cultures were checked by gram-staining and PCR-RFLP (29) for purity. The presence of plasmid insertion in the *P. salmonis* genome was further confirmed by PCR with the Tn7R primer (5′-CACAGCATAACTGGACTGATTTC-3′) that hybridizes with the Tn7R sequence in the plasmid and glmsps2 primer (5′-GCATGAACATCTTGCACCATT-3′) that hybridizes to the 3’-end of *glmS* gene in *P. salmonis* chromosome (Table S1). Positive strains were grown in NPA plus gentamicin and trimethoprim for maintenance and downstream assays. Transconjugants were stored frozen in glycerol at −80°C.

### Conjugative efficiency

The viable number of *P. salmonis* transconjugants was estimated by the most probable number (MPN) method (30). A detailed description of MPN methodology can be found in the supplemental methods. Conjugative efficiency was calculated as the ratio of viable transconjugants grown in NPB supplemented with gentamicin plus trimethoprim (transconjugants) to viable *P. salmonis* cells grown in NPB plus trimethoprim (receptors).

### Plasmid stability assay

Six isolates harboring the Tn7 element from plasmids pJMP2754 or pJMP2774 (*sfGFP*(+) or sgRNA-*sfGFP* strains) were grown for 96 h at 18°C in NPB supplemented with gentamicin and trimethoprim; then, 700 µL of each culture was centrifuged at 6,000 × *g* for 3 min at 4°C and washed twice with NPB to remove the residual antibiotics. Washed cells were inoculated to a final OD_600_ = 0.01 in 700 µL NPB in a 48-well plate without antibiotics and grown for 96 h at 18°C. The procedure of dilution and growth was repeated nine times (approximately 12 generations per growth experiment, a total of 132 generations). Then, retention of the Tn7 element was verified by PCR with primers Tn7R/glmsps2 and *sfGFP* fluorescence.

### *sfGFP* fluorescence measurements

Transconjugant *P. salmonis* strains were grown for 40 h (mid-exponential phase) in NPB supplemented with gentamicin and trimethoprim. Then, 1 mL was centrifuged for 3 min at 6,000 × *g* at 4°C and resuspended in 1 mL of fresh NPB. After resuspension, 200 μL were directly added into sterile black 96-well plates or diluted 1:10, 1:100, or 1:1,000 for fluorescence measurements (excitation 475 nm and emission 500–550 nm) in a GloMax Explorer Multimode Microplate Reader (Promega). Fluorescence measurements were normalized according to the optical density (OD_600_) of the cultures. Additionally, fluorescence of transconjugants carrying Tn7 elements from pJMP2754 (*sfGFP*(+) strain) was detected using confocal microscopy. For this, 10 µL of stationary phase cultures were transferred to a 1% low melting point agarose pad (Lonza) and imaged. Expression of *sfGFP* was visualized using a C2+ Confocal inverted microscope (Nikon) and a 60× objective lens; images were acquired using the NIS-elements program (Nikon).

### Estimation of knockdown efficiency

Four independent transconjugant clones carrying Tn7 elements from pJMP2754 or pJMP2774 (*sfGFP*(+) or sgRNA-*sfGFP* strains) were grown to the stationary phase and then inoculated in 24-well plates to a final OD_600_ = 0.01 in 1.2 mL of NPB, supplemented with or without 10 µM, 100 µM, or 1,000 µM of the inducer IPTG. Plates were incubated for 20, 44, 68, or 92 h at 18°C with shaking, and the cultures were collected at each time point to quantify *sfGFP* fluorescence in a microplate reader as described above. Fluorescence measurements were normalized according to the optical density (OD_600_) of the cultures, with the results expressed as the ratio of sgRNA-*sfGFP* to *sfGFP*(+). GraphPad Prism version 8.0.1 was used for graphical presentation and statistical analysis of the results.

### Growth curve analysis

For growth curve assays, stationary state bacteria (OD_600_ = 1.1–1.3) were used as inoculum. Detailed growth conditions can be found in the supplemental methods. Growth curve parameters were calculated based on raw growth data using the R package Growthcurver (31). Growth parameters were obtained for each biological replicate, and data were then processed in Excel to compare replicates separately. GraphPad Prism version 8.0.1 was used for graphical presentation and statistical analysis of the results.

### Prediction of TnsD binding sites in *P. salmonis* CGR02 and alignment of *attTn7* box in different strains

A MEME motif was created from the terminal region of all coding sequences retrieved from the NCBI Gene database using the query “*glmS*[Gene Name] AND alive[prop] AND glutamine AND fructose AND phosphate” (n = 3,214). This motif was then scanned against the *P. salmonis* CGR02, *E. coli* K-12 (NCBI RefSeq NC_000913.3), and *Proteus mirabilis* HI4320 (NCBI RefSeq NC_010554.1) genomes, using the FIMO tool (32) and filtering at a q-value cutoff of 0.05. The 92 available RefSeq genomes for *P. salmonis* were downloaded from the NCBI website (https://www.ncbi.nlm.nih.gov/datasets/genome/?taxon=1238 accessed on July 23^th^, 2025). For each genome, the sequence of the *glmS* CDS plus 30 nucleotides downstream was extracted using BEDTools v2. 31.1 (33) and aligned with MAFFT v7.526 (34). Finally, a sequence logo of the TnsD binding region (*attTn7* box) was generated using WebLogo (35).

### sgRNA design

The sgRNA spacers were designed as described in Banta *et al*. (https://github.com/ryandward/sgrna_design) (2). Briefly, 20 nucleotide sequences were selected using the following criteria: next to a NGG protospacer adjacent motif (PAM), targeting the non-template strand toward the 5′ end of the gene, and having maximum specificity when aligned to the target genome. Alignment with *P. salmonis* CGR02 genome (NCBI RefSeq assembly GCF_001534725.1) was performed using Bowtie (36). Two oligonucleotides, forward and reverse, were designed to obtain the selected sgRNA when annealed. Each oligonucleotide has an additional 4 bp sequence at the 5’ end that is complementary with the *BsaI* sticky ends that are generated in pJMP2782 plasmid, enabling ligation into the plasmid. Two sgRNA spacers targeting the *fur* gene were designed and named sgRNA-*fur1* and sgRNA-*fur2*.

### Vector construction

Plasmid DNA was extracted from 15 mL *E. coli* BW25141 pJMP2782 culture using the EZNA Plasmid Mini Kit I (Omega Bio-tek) following the manufacturer’s instructions, digested using the *BsaI* restriction endonuclease (New England Biolabs) for 10 h at 37°C, purified using Zymo DNA Clean and Concentrator-5 kit, and quantified using Qubit Fluorometric Quantitation System (Life Technologies). The oligonucleotides were annealed by incubating them at 95°C for 5 min and allowing them to cool to room temperature. This final solution was diluted 40 times. Ligations of the dsDNA oligos to the digested pJMP2782 plasmid were performed with T4 DNA ligase (NEB) in a 10 μL reaction volume with 50 ng of digested pJMP2782 and 100 μM of each oligo. The reaction was incubated for 16 h at 16 °C followed by heat inactivation for 10 min at 65 °C. Reactions were transformed into chemically competent *E. coli* SM10 cells as previously described; after growth in selective media, *P. salmonis* transconjugants (sgRNA-*fur1* and sgRNA-*fur2*) were checked by PCR as mentioned above.

### RNA purification

The procedure for purifying RNA from *P. salmonis* strains sgRNA-*fur1* and sgRNA-*fur2* is available in the supplemental methods.

### Quantitative real-time PCR (qPCR) assays

RT-qPCR reactions were carried out in an AriaMx Pro thermal cycler (Agilent Technologies). cDNAs were synthesized from 1 µg of DNA-free RNA using the High-Capacity RNA-to-cDNA Kit (Applied Biosystems) according to the manufacturer’s instructions. cDNAs were diluted to 5 ng and used as a template for qPCR, with primers designed against the genes of interest (Table S1). For full details on how the PCR conditions and gene expression analysis were carried out, please refer to the supplemental methods.

### RNA sequencing and analysis

Three libraries for control (uninduced sgRNA-*fur1* strain) and three for IPTG-induced sgRNA-*fur1* strain were generated at SeqCenter (Pittsburgh, PA), using the TruSeq Stranded Total RNA Library Prep (Illumina), with bacterial rRNA removal. The libraries were paired-end sequenced on a NovaSeq X Plus instrument (Illumina) with read lengths of 150 bp. The RNA-seq raw data quality was examined using FastQC (37) to detect low-quality reads and Illumina adapters, which were subsequently eliminated using BBduk software (38). The filtered reads were mapped against the *P. salmonis* CGR02 genome (GenBank accession no. GCA_001534725.1) using the STAR v2.7.10b RNA-Seq alignment tool. Reads that failed to map to any gene or mapped to multiple genes were removed before expression analysis. Gene quantification was generated using the quantMode option available at the STAR program. To identify the differentially expressed genes (DEGs), the gene counts matrices were previously filtered by removing genes with counts in less than two of three replicates by condition. Finally, low-expressed genes were discarded using the strategy described in the study by Chen et al. (39).

DESeq2 v1.46.0 package (40) was used to detect DEGs. First, raw count data were used to calculate normalization factors to correct for variations in library depth. Next, gene-specific dispersion values were estimated and refined through the shrinkage of gene-wise dispersion. Finally, we fit a negative binomial model to each gene, followed by a differential expression test using the Wald significance test. All these calculations were carried out using R software v.4.3.1. Plots were constructed using the R package ggplot2 (v.3.4.4) (41). For functional analysis of differentially expressed genes, a compilation of gene ontology (GO) terms for *P. salmonis* CGR02 was generated using eggNOG mapper v2.1.9 (42), and an org.db package was constructed using the R package AnnotationForge v 1.42.2. Then, GO enrichment analysis was carried out using the enrichGO function from the R package clusterProfiler (v.4.8.1) (43). Additionally, homologs of the Virulence Factor Database (VFDB) proteins (downloaded on 21st April 2023) were identified using DIAMOND v2.0.15 (44). *P. salmonis* CGR02 genome was also screened for the genes of iron metabolism with the bioinformatics tool FeGenie (45).

### Prediction of Fur-binding sites

For the identification of potential Fur-binding sites (Fur boxes) in the *P. salmonis* genome, 774 experimentally validated transcription factor binding sites were downloaded from the CollectTF database (46). The sequences were converted to the MEME motif format (47) and scanned against the *P. salmonis* genome using the command-line FIMO tool (32). The parameters of the scanning were first tested using *E. coli* genome and selected when 95% of the already described Fur-binding sites on *E. coli* genome were identified (23). The Markov background DNA model for *P. salmonis* genome was constructed using the fasta-get-markov script from MEME suite. Binding site search was carried out with the following parameters: -alpha 0.1 -oc furBkg0Motif2024 --max-strand -- bgfile psal.mk0.bgk --parse-genomic-coord --thresh 0.0001. Then, sequence logos were created using the R package ggseqlogo v0.2 (48).

### Quantification of intracellular iron and proteins

Exponentially growing *P. salmonis* cells (1 mL) were collected by centrifugation at 8,000 × *g* for 10 min at 4°C. The supernatant was discarded, and the pellet was subsequently washed with PBS 1×, 50 mM glycine, 1 mM EDTA, and milliQ water. Following centrifugation at 10,000 × *g* for 10 min at 4°C, the resulting pellet was resuspended in 125 µL of 65% nitric acid (Merck) and incubated at 62°C overnight to fully dissolve the pellet. Total reflection X-ray fluorescence spectroscopy (TXRF) with gallium as the internal standard was performed using a Bruker S2 PICOFOX, as described by González *et al*. (49). The detailed growth conditions and a detailed description of the protein quantification procedure can be found in the supplemental methods.

## RESULTS

### Mobile-CRISPRi functions in *P. salmonis* strains

**Fig 1:**
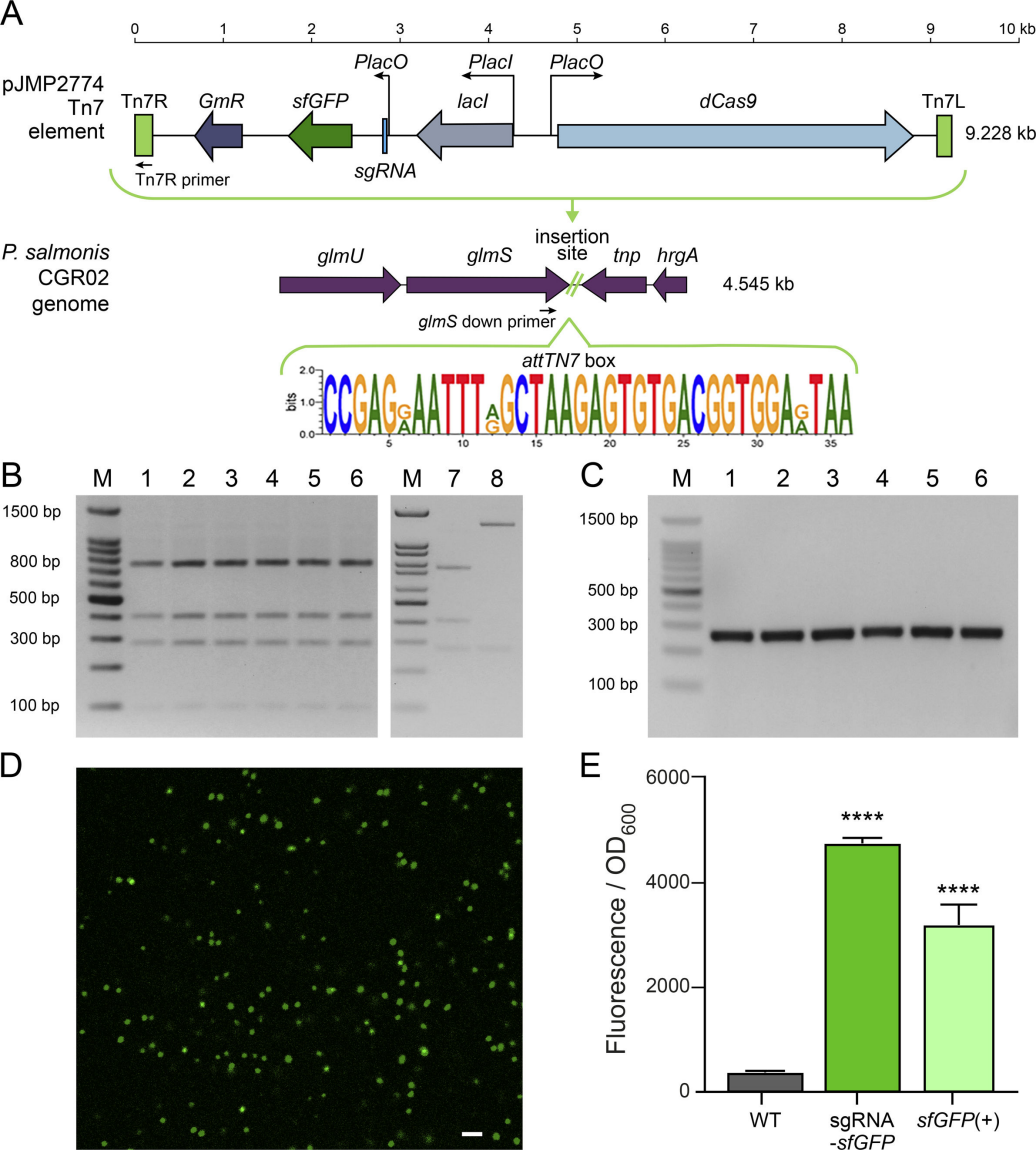
Tn7 integration and functional demonstration of the Mobile-CRISPRi system in *P. salmonis* CGR02. (**A**) Model depicting Tn7 insertion of Mobile-CRISPRi in the genomic context of *P. salmonis* CGR02, and the consensus *attTn7* box (TnsD recognition sequence). The Tn7 element in pJMP2774 contains Tn7R, Tn7L, gentamicin resistance cassette (*GmR*), foreign genes *dcas9*, *sfGFP,* and the gmc6 sgRNA targeting the *sfGFP* gene. Primers Tn7R and glmsps2 down, which detects the upstream junction of the integrated Tn7, are also shown. (**B**) PCR-RFLP digestion pattern (16S rDNA, amplification using primers 27F/1492R) for the sgRNA-*sfGFP* (lanes 1–3) and *sfGFP*(+) (lanes 4–6) transconjugants. Wild-type strain and *E. coli* SM10 are included as controls of PCR-RFLP patterns (lanes 7 and 8, respectively). (**C**) PCR confirmation of Mobile-CRISPRi insertion using primers Tn7R/glmsps2, down shown in (**A**), with an expected fragment of ~300 bp. Lane M indicates the molecular weight standard. (**D**) Confocal microscopy showing the green fluorescence of *sfGFP*(+) strain. Bar = 10 µm. (**E**) The GFP fluorescence of the *P. salmonis* sgRNA-*sfGFP* and *sfGFP*(+) strains in the absence of the IPTG inducer was compared with that of the wild-type strain (WT). Fluorescence was normalized according to the optical density (OD_600_) of each culture. Each bar represents the mean of four biological replicates, while the error bars indicate the standard deviation. (Unpaired *t*-test, ****P < 0.0001).

In order to test the functionality of the Mobile-CRISPRi platform in *P. salmonis*, we used the plasmids pJMP2754 and pJMP2774, which were designed by Banta *et al* (2). The pJMP2754 plasmid contains the “test” version of the Mobile-CRISPRi, which consists of a superfolder green fluorescent protein gene (*sfGFP*) with constitutive expression and an IPTG-inducible *dcas9* gene but lacks the sgRNA. We used this plasmid to produce a control strain named *sfGFP*(+). We used pJMP2774, which contains an IPTG-inducible sgRNA (designated as gmc6) targeting the *sfGFP* gene, to generate the strain sgRNA-*sfGFP*. A schematic representation of the pJMP2774 Mobile-CRISPRi and the predicted insertion site in the genome of *P. salmonis* CGR02 are shown in Fig. 1A.

Previous works have shown that the TnsD protein recognizes and binds to a DNA sequence called *attTn7* box near the end of the highly conserved *glmS* gene and subsequently inserts the transposon downstream of the *glmS* open reading frame (50). In this study, a MEME motif constructed with 3,214 *glmS* sequences was used to scan the *P. salmonis* genome for matches to the TnsD-binding site. This motif was validated using the *E. coli* and *P. mirabilis* genomes for which previous TnsD binding sites have been reported (51, 52). For *P. salmonis*, a single site at the end of *glmS* was identified with a q-value < 0.05 (Table S4). Then, we examined the conservation of the Tn7 transposon site in the genomes of 92 strains of *P. salmonis*, including representatives of the two genogroups described for this species. Consistently, our analysis of the *attTn7* binding site of *P. salmonis* showed that it is highly conserved in the 92 sequences of *P. salmonis* strains (Fig. S1).

**Fig 2:**
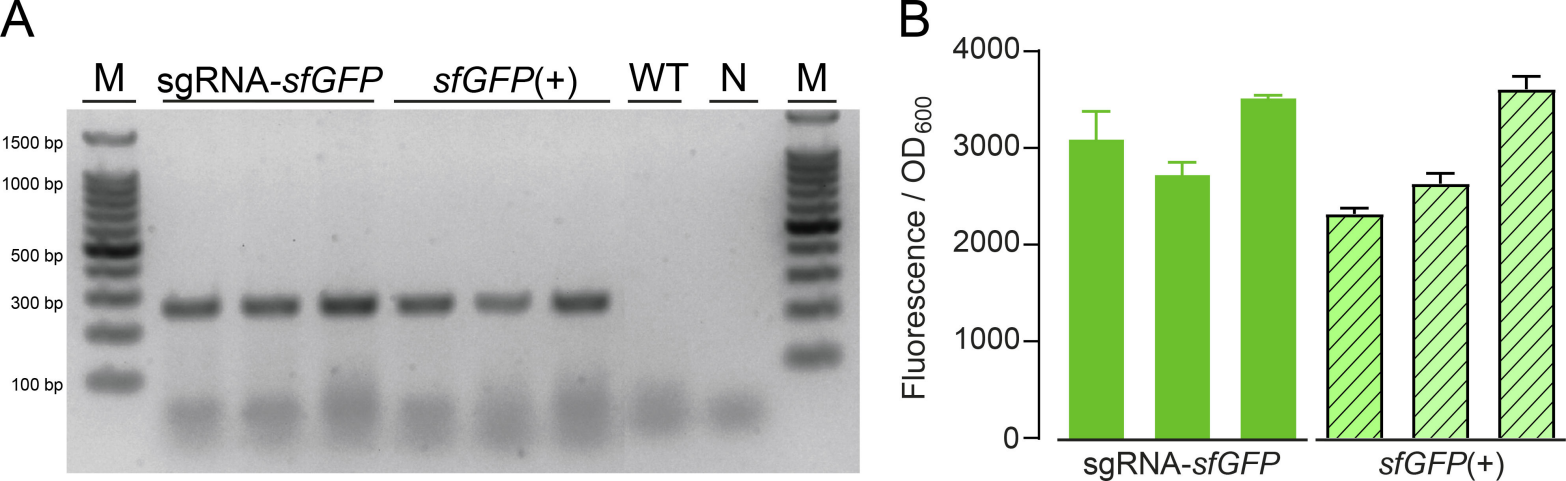
Stability of the Mobile-CRISPRi system in *P. salmonis*. (**A**) The gel electrophoresis shows the PCR product to confirm presence of the insertion using Tn7R/glmsps2-down primers that detect the upstream junction of the integrated Tn7 for six different *P. salmonis* clones harboring the Mobile-CRISPRi system (*sfGFP*(+) or sgRNA-*sfGFP* strains). The wild-type strain (WT) and a PCR-negative control (N) are also included. Lane M shows the molecular weight standard. (**B**) The relative fluorescence of sgRNA-*sfGFP* and *sfGFP*(+) transconjugants was normalized according to the optical density (OD_600_) of each culture, with three biological and three technical replicates in each case. The graph shows three distinct clones of *P. salmonis* transconjugants in the absence of the IPTG inducer.

Considering the fastidious nature of *P. salmonis*, the bacterium was routinely cultured in nutrient media (NPB or NPA) at 18°C. The conjugation experiments were also conducted in these conditions, which allowed the growth of both *P. salmonis* and *E. coli*. To decrease the chance of co-culturing both bacteria after conjugation, transconjugants were selected in NPA medium supplemented with gentamicin (to select for the insertion of the Mobile-CRISPRi module) and with trimethoprim, since *P. salmonis* is naturally resistant to trimethoprim and sensitive to gentamicin (Fig. S2; [53]). In addition, PCR-RFLP assay was performed for every *P. salmonis* transconjugant to ensure its purity (no carry-over of *E. coli* cells, Fig. 1B). Afterward, the correct insertion of the Mobile-CRISPRi module was confirmed by the amplification of the expected ~300 bp sequence. This was obtained using the Tn7R primer and the glmsps2 down primer (Fig. 1A; Table S1) in the transconjugant clones (Fig. 1C). The transfer of Mobile-CRISPRi was measured by quantifying the number of recipient cells (transconjugants). In order to do this, we determined the MPN on gentamicin + trimethoprim NPB medium as a fraction of total *P. salmonis* cells recovered in trimethoprim-supplemented NPB. *P. salmonis* CGR02 showed transfer efficiencies ranging from 1.00 × 10^−1^ to 2.57 × 10^−1^ (Table S5), depending on the experiment. Bright green fluorescence of *P. salmonis* transconjugants harboring the *sfGFP* gene (*sfGFP*(+)) was detected by confocal microscopy (Fig. 1D). Furthermore, in relation to the wild-type (WT) strain, a significant increase in fluorescence was detected in the transconjugant strains harboring the Mobile-CRISPRi system without *dcas9* and gmc6 sgRNA induction (Fig. 1E). The inserted Mobile-CRISPRi module remained stable in the *P. salmonis* CGR02 genomic context. Following 10 subcultures in the absence of antibiotic selection pressure, the transconjugants retained the inserted module (Fig. 2A). Furthermore, following over 130 generations, *P. salmonis* strains sgRNA-*sfGFP* and *sfGFP*(+) retained the ability to emit fluorescence (Fig. 2B).

**Fig 3:**
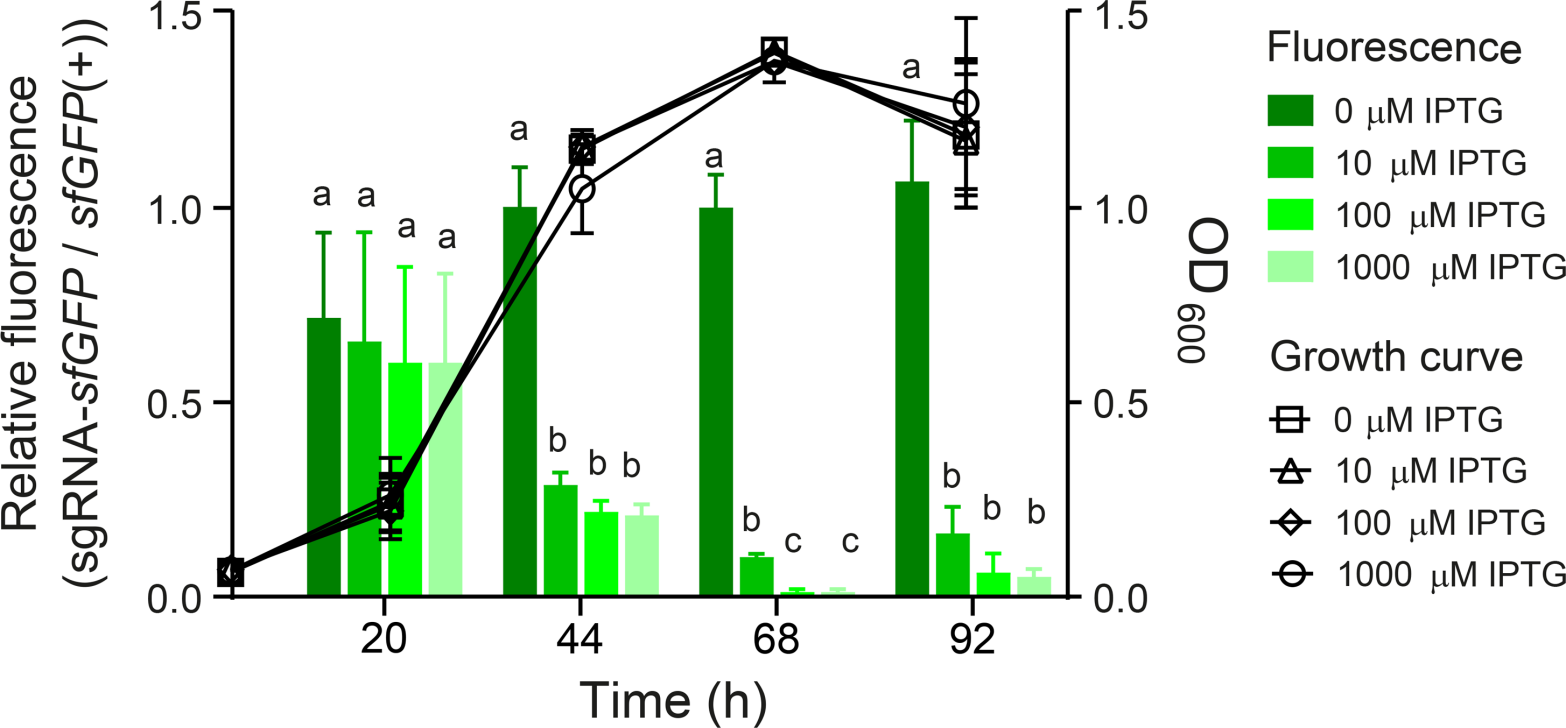
Silencing of *sfGFP* expression via Mobile-CRISPRi in *P. salmonis* CGR02. The green bars represent the relative fluorescence (fluorescence measurements normalized by OD_600_) of the inducible sgRNA-*sfGFP* strain, divided by the fluorescence of the *sfGFP*(+) strain at different time points with increasing IPTG concentrations (0 µM, 10 µM, 100 µM, or 1,000 µM). Each data point is based on three biological replicates. The black symbols show the growth curve of *P. salmonis* harboring sgRNA-*sfGFP* with different concentrations of IPTG. Left Y axis shows relative fluorescence, and the right Y axis shows optical density (OD_600_). Letters over the bar graphs indicate statistical differences (evaluated by one-way ANOVA for each time point and Tukey multiple comparisons post-test).

To demonstrate the efficacy of the silencing system in the genetic context of *P. salmonis*, the expression of *dcas9* and the sgRNA targeting *sfGFP* was induced using different concentrations of IPTG. As shown in Fig. 3, the relative fluorescence decreased in the presence of the inducer. In subsequent experiments, 100 µM of IPTG was used, as it was the lowest concentration with the greatest effect, exhibiting a knockdown efficiency of 98.7% at 68 h, which was similar to that of 1,000 µM (98.7%), but significantly higher than that of 10 µM of IPTG (89.7%). Furthermore, the induction of *dcas9* in conjunction with the *sfGFP* targeting sgRNA, or the increasing concentrations of IPTG, did not result in any alterations to the growth of the *P. salmonis* transconjugants in NPB (Fig. 3).

**Fig 4:**
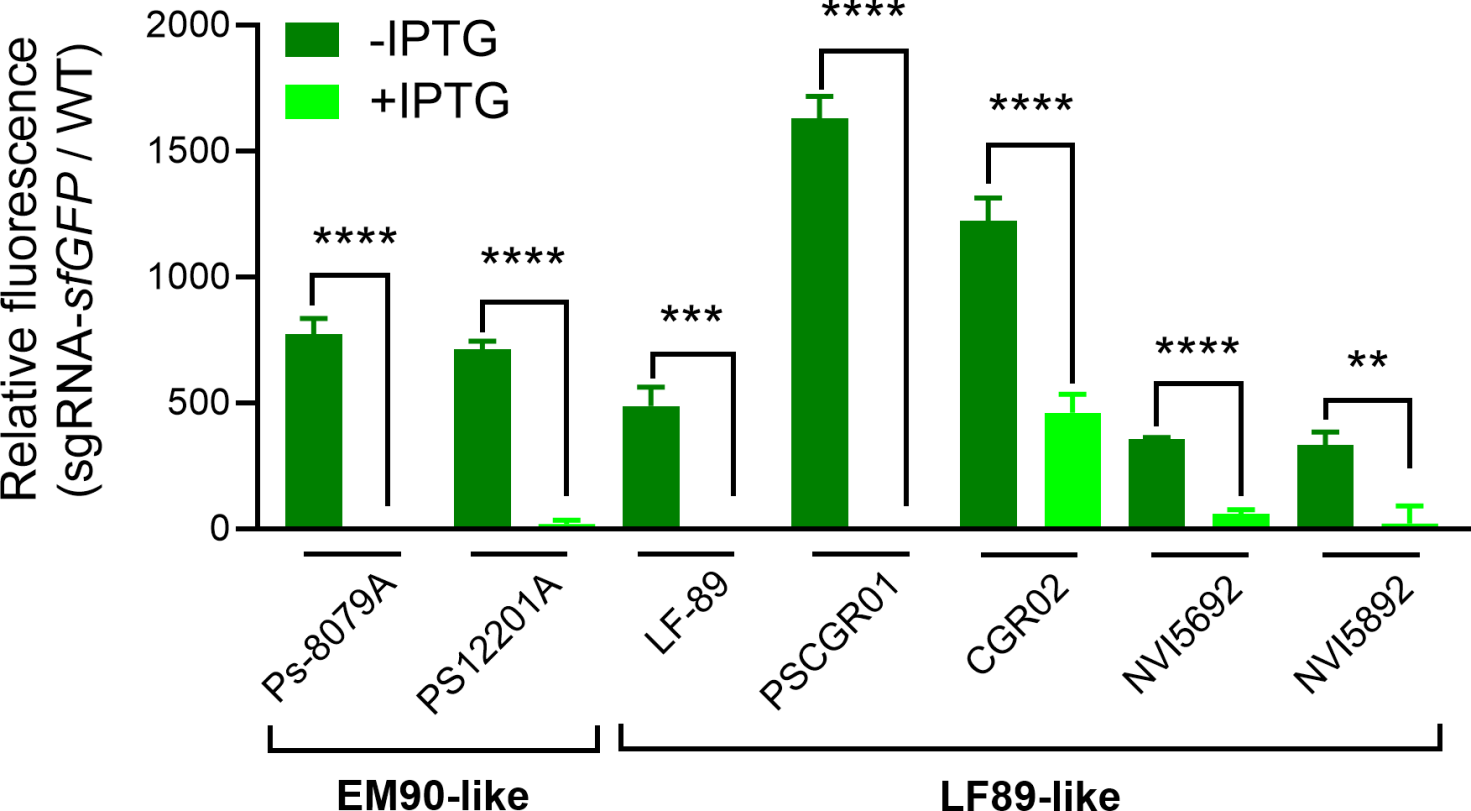
Fluorescence of different *P. salmonis* strains carrying the Tn7 element from the Mobile-CRISPRi silencing system. The genogroup of each strain is indicated below the names. The green bars show the relative fluorescence of the different *P. salmonis* strains, which were normalized by the optical density of the culture (OD_600_) and divided by the normalized fluorescence of the wild-type strain. Three biological replicates of each *P. salmonis* strain are shown, with or without supplementation of the inducer IPTG (100 µM). Statistical significance was evaluated using an unpaired *t*-test comparing each strain with its control without IPTG. (**P < 0.01, ***P < 0.001, ****P < 0.0001).

In order to provide further evidence for the broad application of the Mobile-CRISPRi silencing system in *P. salmonis*, the system was applied to two strains of genogroup EM90-like (*P. salmonis* 12,201 and 8,079) and four strains of genogroup LF89-like. Of these, two were isolated in Chile (*P. salmonis* LF-89 [ATCC VR-1361] and PSCGR01), one was a Norwegian strain (NVI5692), and one was a Canadian strain (NVI5892). All strains (Table S2) were transformed using the pJMP2774 plasmid (Table S4). As a result, all strains tested were able to conjugate with *E. coli,* and all transconjugants contained the inserted Mobile-CRISPRi module, with transfer efficiencies ranging from 1.00 × 10^−7^ for the LF-89 strain to 1.00×2.57 × 10^−1^ for the PS12201A and the CGR02 strains (Table S5). Moreover, all the strains were able to express fluorescence and to silence *sfGFP* expression in the presence of IPTG (Fig. 4).

### Mobile-CRISPRi targets the *fur* gene

**Fig 5:**
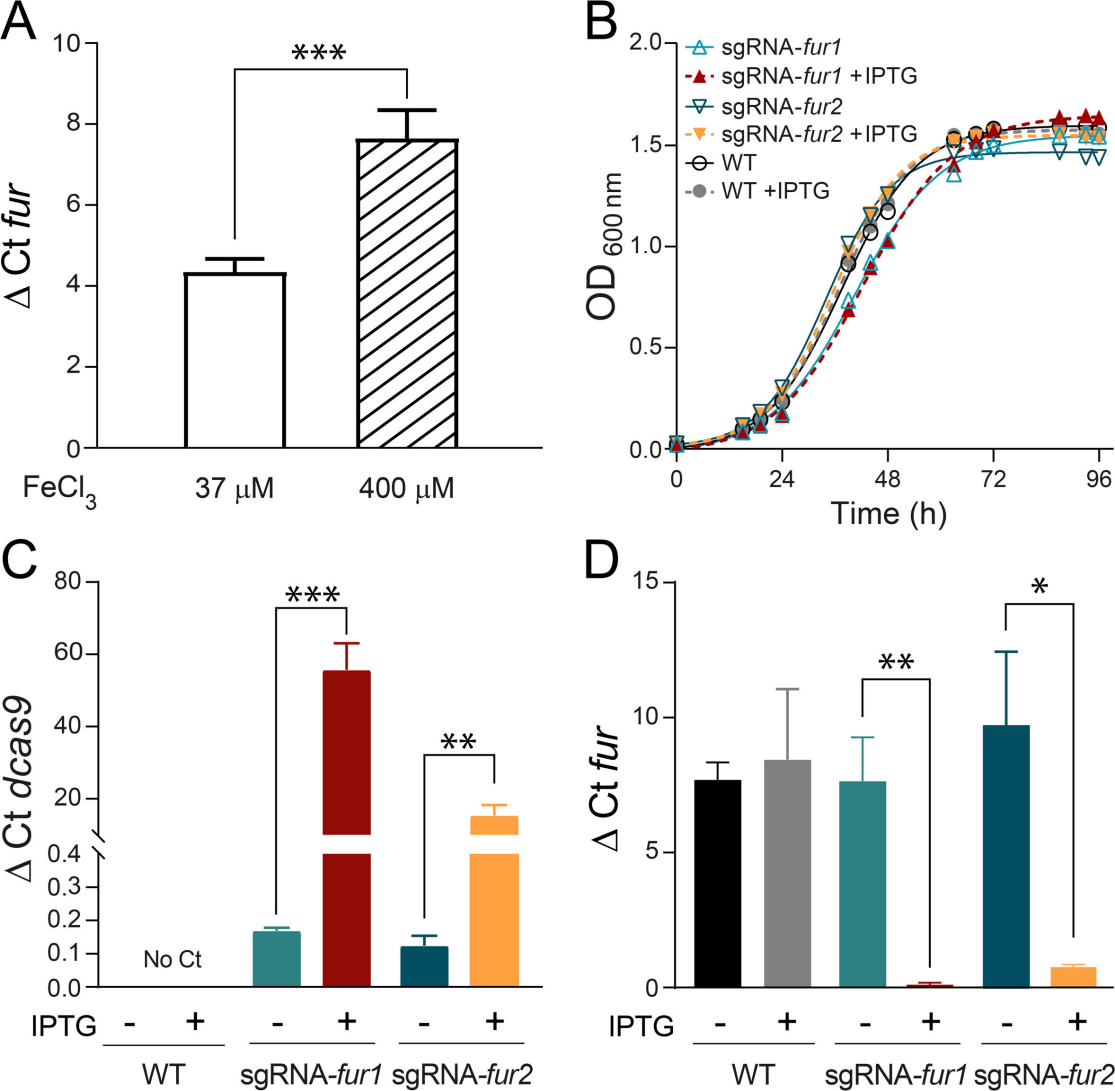
Silencing of *fur* expression via Mobile-CRISPRi in *P. salmonis* CGR02. (**A**) *fur* expression in the WT strain grown in culture media supplemented with 37 µM (*n* = 3) or 400 µM FeCl_3_ (*n* = 5). Expression levels (ΔCt) were normalized by the housekeeping gene *recF* (unpaired *t*-test, ***P < 0.001). (**B**) Growth curves of *P. salmonis* WT, sgRNA-*fur1,* and sgRNA-*fur2* strains with or without IPTG supplementation (*n* = 3). (**C**) Expression of *dcas9* (ΔCt) normalized by *recF* gene in WT, sgRNA-*fur1,* and sgRNA-*fur2* strains (*n* = 3). (**D**) *fur* expression in WT, sgRNA-*fur1,* and sgRNA-*fur2* strains is shown as ΔCt values, normalized by the *recF* gene (*n* = 5 for WT, *n* = 3 for sgRNA-fur1 and sgRNA-fur2 strains). In panels C and D, statistical significance was evaluated using an unpaired *t*-test with Bonferroni correction to compare each strain with its respective control, which was grown without IPTG. (*P < 0.05, **P < 0.01, ***P < 0.001).

To further test the utility of the Mobile-CRISPRi system, two Mobile-CRISPRi variants were constructed containing sgRNAs targeting the *fur* gene of *P. salmonis* (54). This gene encodes a monocistronic mRNA, which makes the polar effect of CRISPRi unlikely. All the analyses of gene expression and growth curves of the *fur* knockdown RNA strains (sgRNA-*fur1* and sgRNA-*fur2*), induced and uninduced by IPTG, were carried out in the NPB medium supplemented with 400 μM FeCl_3_. This iron concentration increased the relative expression of *fur*, as measured by RT-qPCR (Fig. 5A), without negatively affecting bacterial growth (Fig. 5B). A closer inspection of the growth curve parameters revealed statistically significant differences in the maximum load and the area under the curve (AUC) for the sgRNA-*fur1* and sgRNA-*fur2* strains when compared with the WT strain, although these changes were less than 9.3% for each parameter in both strains (Fig. S3). It is interesting to note that the doubling time appeared to be strain-dependent, as it remained consistent when comparing induced and uninduced curves for the same strain. Although this parameter was similar for all strains, the generational time for sgRNA-*fur1* was 20% higher than the WT and 24.6% higher than the sgRNA-*fur2* strain in the presence of IPTG. In contrast, it did not change significantly between the sgRNA-*fur2* and the WT strain, suggesting that the sgRNA-fur1 insertion had a moderate but significant impact on the strain’s replication time.

The expression of *dcas9* in sgRNA-*fur1* and sgRNA-*fur2* strains was analyzed by RT-qPCR in the absence or presence of IPTG induction. Basal levels of *dcas9* expression were detected in the uninduced strains, and a significant increase in *dcas9* expression was observed upon induction of sgRNA-*fur1* and sgRNA-*fur2* strains with IPTG (Fig. 5C). We then characterized Mobile-CRISPRi efficiency to silence the endogenous *fur* gene by RT-qPCR. As expected, the levels of *fur* mRNA were significantly decreased in the presence of IPTG, both in sgRNA-*fur1* and sgRNA-*fur2* strains (Fig. 5D). IPTG induction of *dcas9* and sgRNA expression for 40 h in sgRNA-*fur1* and sgRNA-*fur2* strains depleted *fur* mRNA by at least 95% of the levels measured in the corresponding uninduced or WT strains (Fig. 5D). As demonstrated in Fig. 5B and Fig. S4, the induction of *dcas*9 and sgRNA expressions at a previously defined concentration of 100 µM IPTG did not result in a significant growth defect, showing no differences in the doubling time for the sgRNA-*fur1* and sgRNA-*fur2* strains when IPTG is added. Furthermore, both strains exhibited an increase in maximum load of 6.2% and 5.8%, respectively. The area under the curve increased to 84.6 and 91.7, respectively, in comparison to 82.4 and 89.4 for the same strains without IPTG induction. This indicates that the observed effects are not due to toxic effects resulting from overproduction of dCas9, as has been previously observed in other bacteria (55, 56). This result also indicates that the knockdown of *fur* does not have a detrimental effect on the viability of *P. salmonis* during growth in NBP.

### RNA-seq characterization of CRISPRi-mediated *fur* knockdown

**Fig 6:**
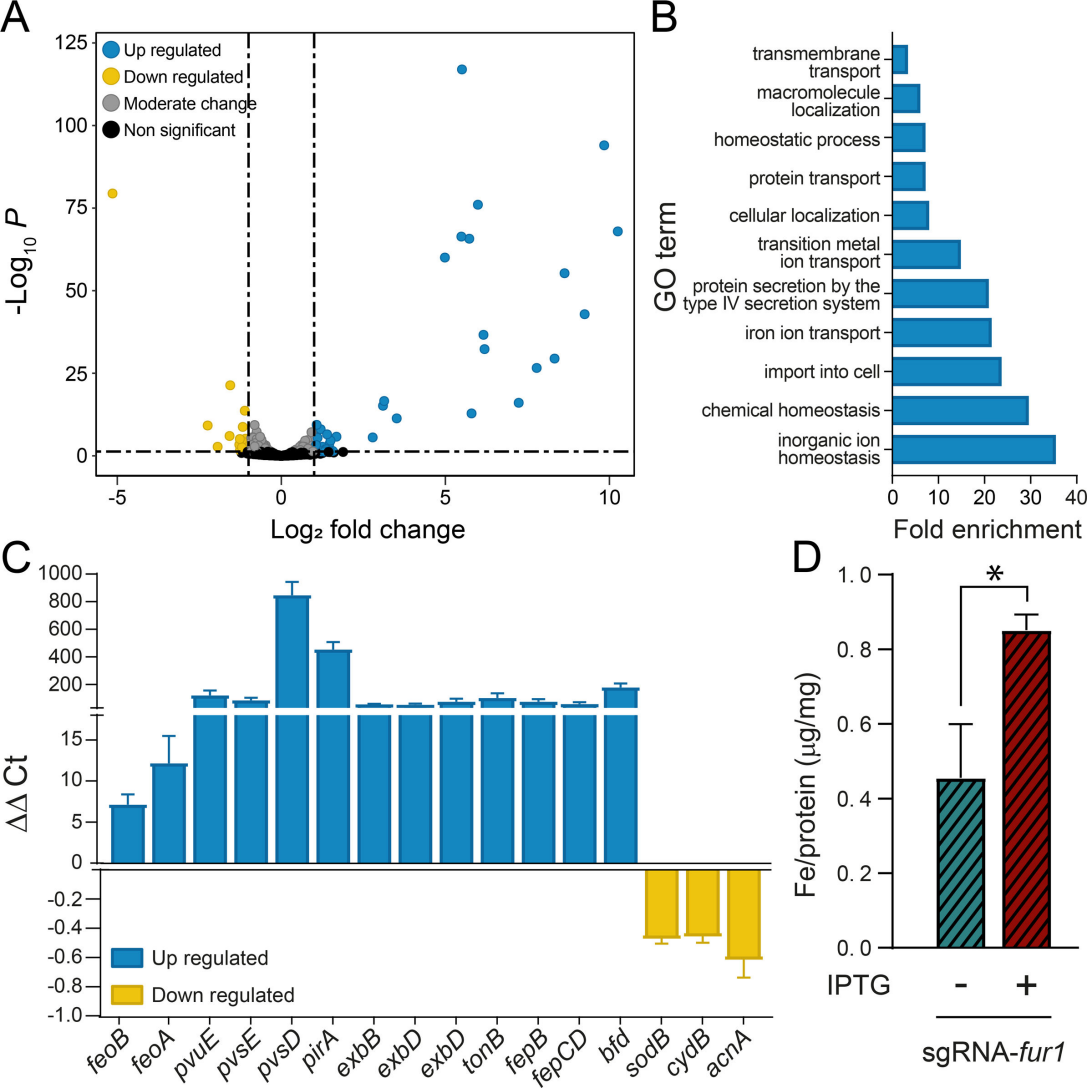
Comparative transcriptomic analysis of *fur*-knockdown in *P. salmonis* CGR02. (**A**) Volcano plots from the DESeq2 analysis of differentially expressed genes between *P. salmonis* sgRNA-*fur1* knockdown strain (*n* = 3 biological replicates) and the isogenic strain without IPTG inducer (control, *n* = 3 biological replicates). The blue dots (*n* = 45) represent significantly upregulated genes, the yellow dots (*n* = 24) represent significantly downregulated genes (adjusted *P*-value <0.05, |log_2_FC| > 1). Gray dots represent no significant change of expression. (**B**) GO enrichment analysis of upregulated genes. List of significantly overrepresented GO terms in the DEGs (log_2_ FC > 1) in the sgRNA-*fur1* knockdown strain. (**C**) RT-qPCR quantification of transcripts for the most up- or down-regulated genes identified in the RNA-seq analysis. Expression levels of *P. salmonis* genes are expressed as ΔΔCt values of the sgRNA-*fur1* knockdown strain normalized by the control, and *recF* as housekeeping gene. Increased gene expression relative to control bacteria is shown in blue, and decreased expression in yellow (*n* = 3 biological replicates). (**D**) Intracellular iron concentration in sgRNA-*fur1* strain with (+) or without (-) the inducer IPTG (unpaired *t*-test, *P < 0.05, *n* = 3 biological replicates).

To elucidate the effects of *fur* knockdown on global gene expression, we performed RNA-seq using the IPTG-induced and -uninduced sgRNA-*fur1* strains under iron-replete conditions (Fig. 6). The repressive regulatory effects exerted by Fur in wild-type bacteria under iron-replete conditions should be alleviated due to the knockdown of *fur* in the IPTG-induced strain. RNA samples were prepared from three biological replicates of the exponentially growing sgRNA-*fur1* strain after two passages in NPB plus 400 μM FeCl3, with and without IPTG. In these conditions, the depletion of *fur* was found to be significantly higher than that observed after a single passage in broth supplemented with IPTG (Fig. S4). The RNA-seq data were analyzed for accuracy (adjusted *P*-value < 0.05), revealing 219 differentially expressed genes. Filtering by fold-change (|log_2_FC| > 1; see Table S6), we found that the expression level of 69 genes (45 upregulated and 24 downregulated) changed significantly between the IPTG-induced and uninduced sgRNA-*fur1* strains (Fig. 6A).

From Table S6, it becomes evident that the genes with the highest fold change were those upregulated in the IPTG-induced sgRNA-*fur1* strain. Moreover, the number of genes upregulated was higher (66%) than the genes downregulated (34%) in the knockdown strain. GO enrichment analysis indicated that the biological processes “inorganic ion homeostasis,” “import into cell,” “iron ion transport,” and “secretion by the type IV secretion system” were enriched among the genes upregulated in the sgRNA-*fur1* strain (adj. *P*-value < 0.05, log_2_ FC > 2; Fig. 6B); however, no enriched biological process was detected for downregulated genes. Among the most highly expressed genes in the induced sgRNA-*fur1* strain (*N* = 18, adjusted *P*-value < 0.05, log_2_ FC > 2, Table 1), we identified genes encoding putative components of the FeO and FeP systems, which are involved in ferric and ferrous transport, respectively (57). We also identified the *bfd* gene, which encodes a bacterioferritin-associated ferredoxin required for mobilizing iron stored in bacterioferritin (58). The highest fold inductions in the knockdown strain were observed for a gene encoding a putative TonB-dependent siderophore receptor (PirA), members of the TonB-ExbB-ExbD energy transduction apparatus (59), and genes encoding components of siderophore synthesis and transport (Table 1). To gain insight into the overall iron metabolism potential of *P. salmonis*, the bioinformatic tool FeGenie was used to identify genes coding for proteins involved in iron homeostasis. The results revealed that 23 genes related to iron transport, siderophore synthesis, siderophore transport, and gene regulation are present in the *P. salmonis* genome, 16 of them were upregulated (log_2_ FC > 2) in the sgRNA-*fur1* strain (Table S6).

**TABLE 1:** Iron homeostasis genes upregulated in the knockdown strain^*a*^

Gene ID	Log_2_FC	Gene name	Product description	Conserved domains	Predicted function
AWJ11_02085	3.52	*feoC*	FeoC-like transcriptional regulator	Transcriptional regulator HTH-type	Fe^2+^ transport
AWJ11_02090	3.13	*feoB*	Transporter of a GTP-driven Fe^2+^ uptake system	Ferrous iron transport protein B/ nucleoside recognition	Fe^2+^ transport
AWJ11_02095	2.78	*feoA*	Fe^2+^ transport system protein A	FeoA domain	Fe^2+^ transport
AWJ11_06980	6.19	*pvuE*	ABC transporter family protein	ABC-type cobalamin/Fe^3+^-siderophores transport system, ATPase component	Siderophore uptake
AWJ11_06985	6.16	*pvsE*	PLP-dependent decarboxylase	Pyridoxal-dependent decarboxylase, C-terminal sheet domain	Siderophore biosynthesis
AWJ11_06990	8.63	*pvsD*	IucA/IucC family protein	IucA / IucC family; ferric iron reductase FhuF-like transporter	Siderophore biosynthesis
AWJ11_06995	8.33	*pvsC*	MFS transporter	Arabinose efflux permease, multidrug efflux MFS transporter	Siderophore export
AWJ11_07000	9.24	*pvsD*	IucA/IucC family protein	IucA / IucC family; ferric iron reductase FhuF-like transporter	Siderophore biosynthesis
AWJ11_07005	9.84	*pvsA*	ATP-dependent carboxylate-amine ligase	ATP-grasp domain	Siderophore biosynthesis
AWJ11_07010	10.26	*pirA*	TonB-dependent siderophore receptor	TonB-dependent receptor plug domain; TonB-dependent receptor-like, beta-barrel	Siderophore uptake
AWJ11_07015	5.73	*exbB*	Biopolymer transporter ExbB	MotA/TolQ/ExbB proton channel family	Siderophore uptake
AWJ11_07020	5.99	*exbD*	Biopolymer transporter ExbD	Biopolymer transport protein ExbD/TolR	Siderophore uptake
AWJ11_07025	5.80	*exbD*	Biopolymer transporter ExbD	Biopolymer transport protein ExbD/TolR	Siderophore uptake
AWJ11_07030	7.78	*tonB*	Energy transducer TonB	Gram-negative bacterial TonB protein C-terminal	Siderophore uptake
AWJ11_07035	5.51	*fepB*	Iron-siderophore ABC transporter substrate-binding protein	Periplasmic binding protein	Siderophore uptake
AWJ11_07040	5.49	*fepCD*	Iron ABC transporter permease	FecCD transport family	Siderophore uptake
AWJ11_07380	7.24	*bfd*	(2Fe-2S)-binding protein	BFD-like [2Fe-2S] binding domain	Iron mobilization

^*a*^ List of genes with significantly increased levels in the strain with induced expression of the silencing Mobile-CRISPRi system. Differentially expressed genes were grouped in three regions according to their location in *P. salmonis* genome.

In addition, small but significant changes in the expression of the downregulated genes were detected in the IPTG-induced sgRNA-*fur1* strain compared with the uninduced strain, with only 24 genes, including the *fur* gene, showing a log_2_ fold change ≤ −1 (Table S6). Among them, we found genes encoding Fe-S-, heme-, or iron-containing proteins such as *sodB*, *thiC*, *cydB*, *nrdAB*, and *acnA* (Table S6). Validation of the expression profiles of representative up- and down-regulated genes in the IPTG-induced sgRNA-*fur1* strain using RT-qPCR confirmed the expression changes determined by RNA-seq (Fig. 6C). Furthermore, RT-qPCR analysis of a second strain, sgRNA-*fur2*, induced with IPTG, provided further validation of the changes in gene expression resulting from *fur* knockdown (Fig. S5). Consistent with the increased expression of iron acquisition genes, TXRF analysis revealed a significant rise in the intracellular iron concentration of the IPTG-induced sgRNA-*fur1* strain compared with the uninduced strain (Fig. 6D).

### Bioinformatics prediction of Fur-binding sites in upregulated genes

**Fig 7:**
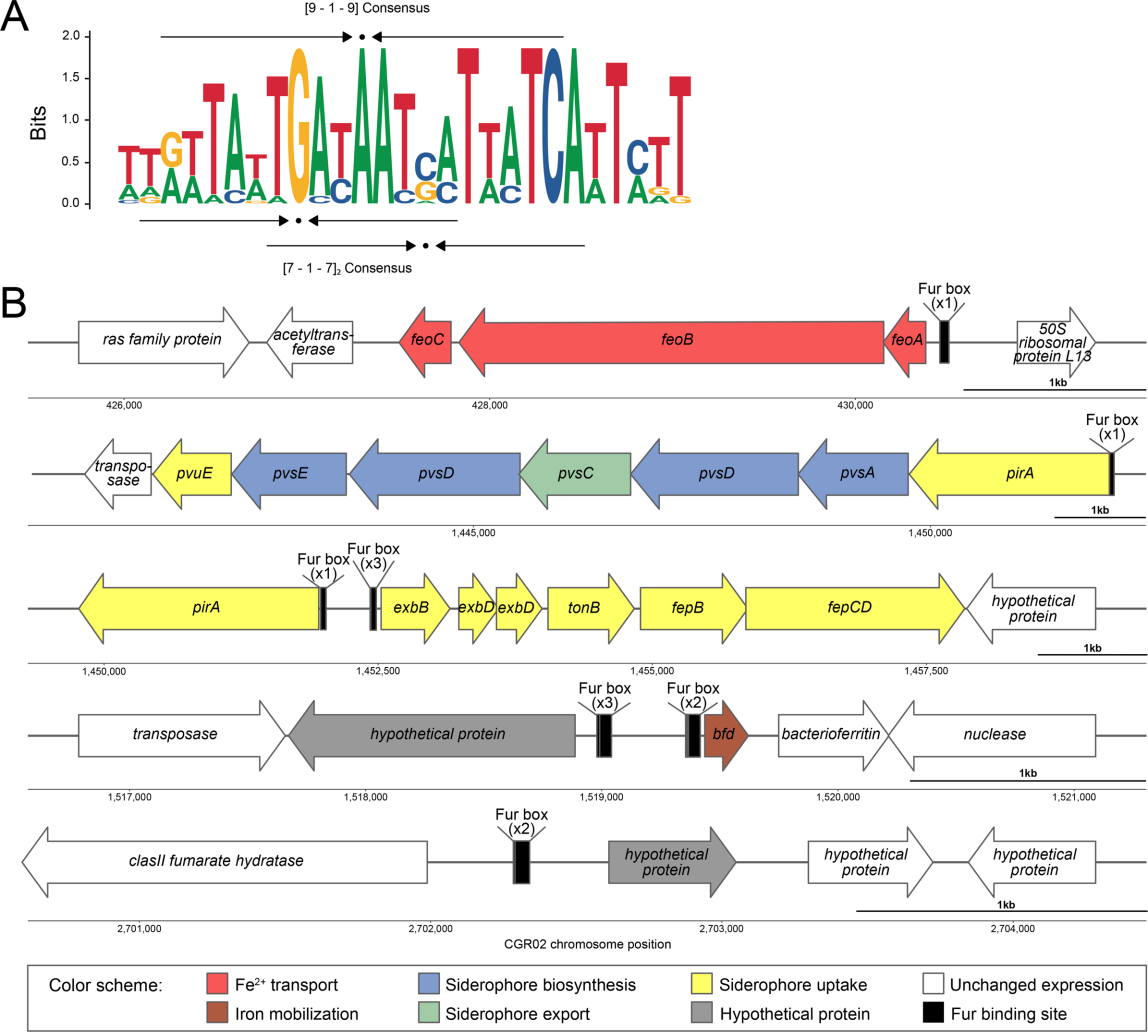
Fur-regulated genes in *P. salmonis* CGR02. (**A**) Logo of Fur box. The image shows the consensus logo for the Fur transcription factor binding sites identified upstream of genes that were upregulated by IPTG induction. The consensus fits the two-dimer model (9-1-9, top arrows), as well as the repeated heptamer model (7-1-7, bottom arrows) for fur binding. (**B**) Schematic representation of the upregulated genes shown in Table 1. Predicted Fur boxes are indicated in black upstream of the regulated gene(s).

Using the *P. salmonis* CGR02 genome, we examined the intergenic regions upstream of upregulated genes to identify sequences corresponding to the Fur (Fur box)-binding site, based on experimentally validated sites from the CollectTF database (46). We found 16 potential binding sites within the 45 genes with log_2_ FC ≥ 1 in the IPTG-induced sgRNA-*fur1* strain (Table S6). Fifteen of these sites correspond to binding sites upstream of genes with log_2_ FC ≥ 2. Orphan Fur boxes were identified in two regions: 77 pb upstream of the gene *feoA,* in the FeO cluster, and 41 pb upstream of a hypothetical protein with a methyltransferase domain gene (AWJ11_07530). In the remaining regions, there are clusters of Fur boxes, consisting of two to four sites. For example, four binding sites can be found upstream of genes from the PirA-PvsACDE-PvuE cluster, and three sites were identified upstream of genes from members of the TonB-ExbB-ExbD energy transduction system. Also, a single unit of transcription as the (2Fe-2S)-binding protein gene *bfd* and the gene AWJ11_07375 exhibit two and three Fur boxes, respectively (Fig. 7). We align the sequences of the 17 binding sites and identify a 9-1-9 bp inverted repeat sequence compatible with the classic Fur box model GATAATGATAATCATTATC described in *E. coli* (54) or the two overlapping 7-1-7 TGATAAT sequence, described in *B. subtilis* (60).

## DISCUSSION

### Implementation of the Mobile-CRISPRi system in the fish pathogen *P. salmonis*

*P. salmonis* is a major salmon pathogen with worldwide distribution and the principal cause of infection-related deaths in the Chilean salmon industry, having an important economic effect in the second largest exporter of salmon after Norway. The use of genetic tools to study non-model organisms, such as the fastidious intracellular pathogen *P. salmonis* (61), is essential to understanding gene function and furthering our knowledge of bacterial pathogenesis and disease. To date, only one gene manipulation technique has been proven to work for homologous recombination in *P. salmonis* (62); however, this approach is complex and time-consuming. Conversely, the CRISPRi gene knockdown editing system is a more advanced synthetic biology tool that is widely used in many microorganisms due to its simplicity and tunability, which can be achieved by optimizing the inducible expression of its components (2–4). Here, we took advantage of the Mobile-CRISPRi system described by Banta *et al*. (2) to achieve reversible and inducible knockdown of target genes in *P. salmonis*. We employed an exogenous *sfGFP* gene, encoding a fluorescent marker protein, to assess the functionality of the silencing system in this bacterium and an endogenous *P. salmonis* gene to further explore the applicability of this methodology. The introduction of the inactivating element by conjugation of a suicide vector in *P. salmonis* was successful in inserting the Tn7 element into the *attTn7* box downstream of the *glmS* gene (as expected in Gamma-proteobacteria), which was highly conserved for the 92 *P*. *salmonis* genomes publicly available. We proved that the Mobile-CRISPRi system was expressed in the *P. salmonis* genetic context and conferred gentamicin resistance and fluorescence to the receptor strain. We also observed basal *dcas9* expression levels in uninduced cells, and an increment of over 20-fold to 60-fold in expression after induction with IPTG. This increment did not impact the growth of *P. salmonis* strains; in fact, they grew to a higher max load and maintained their generational time (Fig. 3 and 5C; Fig. S3). The leaky nature of *dcas9* expression has been reported previously (63), but in our experiments, this basal dCas9 level did not decrease the fluorescence levels as observed by comparing the fluorescence of the *P. salmonis* CGR02 *sfGFP*(+) strain (with constitutive expression of *sfGFP* in absence of the sgRNA) with the fluorescence from the *P. salmonis* sgRNA-*sfGFP* strain (strain harboring the constitutively expressed *sfGFP* gene, and inducible *dcas9* and sgRNA) in the absence of the inducer (Fig. 1E and 2B). As demonstrated in Fig. 2, the stability of Mobile-CRISPRi in the absence of selection was confirmed after over 130 generations. This finding lends support to the proposition that this system could serve as a valuable tool for *in vivo* assays to study host-pathogen interactions or other conditions where the presence of antibiotics is not suitable. Furthermore, not only was the Mobile-CRISPRi system inserted into the expected location and conferred the anticipated phenotypes to *P. salmonis*, but it also successfully silenced the expression of the target *sfGFP* gene in several *P. salmonis* strains from both genogroups, thus supporting its potential broad applicability. However, it is important to note that the intensity of fluorescence varied among the different strains. Specifically, strains from genogroup EM90-like (12,201A and 8079A) and those from Norway and Canada (NVI 5692 and NVI 5892, respectively) exhibited reduced fluorescence compared with genogroup LF89-like strains, particularly CGR02 and PSCGR01 (Fig. 4). This suggests the possibility of differences in gene expression, protein translation, and/or codon usage between these strains. In this regard, a recent analysis revealed substantial rearrangements of DNA segments within 73 *P*. *salmonis* genomes (64). These rearrangements have the potential to disrupt gene-coding and *cis*-regulatory regions, which can greatly influence gene expression and the manifestation of strain characteristics (65). Genomic rearrangements are most likely driven by the action of transposases (66), which are highly abundant and diverse among *P. salmonis* genomes (64). Transposition activity enables bacterial genomes to carry multiple copies of a specific insertion sequence (IS) element, and recombination between IS elements is a prevalent cause of genomic rearrangements. The process of IS expansion also has the potential to lead to gene inactivation and modulation of neighboring gene expression (67, 68). This phenomenon may contribute to the observed variation in phenotype among different *P. salmonis* strains, including the varying conjugation frequency observed between strains (Table S5).

### Silencing *fur* expression in *P. salmonis*

*P. salmonis* can utilize both ferric and ferrous iron, as well as synthesize siderophores, under iron-restricted conditions (69). This suggests that the bacterium possesses various iron transport systems. In line with this, previous bioinformatics analysis has shown that the LF-89 strain of *P. salmonis* harbors a number of iron transport systems, as well as a gene that encodes a Fur homolog (27). This gene was subsequently characterized functionally through heterologous expression in a *Salmonella* Typhimurium Δ*fur* strain (70). In this study, we examined the impact of inactivating *P. salmonis* Fur under conditions of moderately elevated intracellular iron concentration that did not affect bacterial viability using the Mobile-CRISPRi system in combination with transcriptomics. Our results indicate that in the sgRNA-*fur1* strain, genes involved in iron acquisition are no longer regulated by iron and are highly expressed even under conditions of iron supplementation.

In many bacteria, Fur negatively regulates the production and transport of siderophores (19). Consistent with this, upregulation of 13 genes predicted to be involved in siderophore synthesis and transport (Table 1) was detected in the sgRNA-*fur1* strain. The high level of differential expression of genes encoding a TonB-dependent siderophore receptor and putative siderophore biosynthetic proteins in the knockdown strain suggests that Fe³^+^ predominates during *P. salmonis* infection cycles. Consistently, previous studies have shown that *P. salmonis* can use ferric iron in both *in vitro* and *in vivo* infection models. This was evidenced by a decline in growth and enhanced resistance to *P. salmonis* infection following exposure to the ferric iron chelator deferoxamine (71). Our transcriptomic data also suggest that *P. salmonis* has an additional method of iron acquisition: FeoB-mediated Fe²^+^ uptake. In line with this, the evidence indicates that *P. salmonis* resides in a specialized phagosome within the host cell (72). Consequently, a decrease in the pH of the phagosome may result in an increase in the amount of soluble ferrous iron available for uptake through the FeoAB system (73). In this sense, *Legionella pneumophila*, which is phylogenetically related to *P. salmonis* (74), requires the function of FeoB when growing in low-iron conditions or inside the host cells (75). On the other hand, the upregulation of the *bfd* gene is consistent with reports on other bacterial pathogens under iron-starved conditions. The observed upregulation suggests a requirement for the release of iron stored in bacterioferritin (76–78).

It is known that iron-complexed Fur can also act indirectly in the regulation of some genes by repressing the expression of RyhB small RNA (18). The function of RyhB is to repress non-essential proteins that use iron as a way of preserving the iron levels in the cell at low iron conditions (17). Interestingly, among downregulated genes, we identified genes encoding Fe-S-, heme-, or iron-containing proteins such as SodB, ThiC, CydB, NrdAB, and AcnA, suggesting that these genes might be targets of RyhB. Indeed, the genes *sodB*, *cydB,* and *acnA* are recognized targets of RyhB in *E. coli* (17). To date, however, the presence of a RyhB homolog has not been reported in *P. salmonis*.

Our research also indicates that the regulation of overexpressed genes in *fur* knockdown occurs through previously characterized high-affinity sites for the Fur transcription factor (79). An analysis of the binding logo sequence indicates that the regulation of this gene can be achieved through a two-dimer of 5′-GATAAT-3′−5' with each dimer overlapping on opposite sides of the DNA helix (F-F-x-R-R), comprising the 19-bp consensus binding site (80).

Besides gene clusters encoding iron acquisition and mobilization proteins, a number of other genes, mainly components of the type IV secretion system and hypothetical proteins, were also upregulated in the IPTG-induced sgRNA-*fur1* strain, although with smaller changes in expression. Since consensus binding sites for Fur were not predicted within their intergenic regions, the observed differential expression might imply that other regulators may respond to iron concentrations within the cell. Possible examples of these regulators include Irr and RirA (81), neither of which are annotated in the genome of *P. salmonis*. Thus, it appears that other iron-responsive regulators may be present in *P. salmonis* but have not yet been identified.

Although there are a number of technical considerations to take into account when selecting a knockdown method, such as transcript stability and transcript and protein half-life, the Mobile-CRISPRi system described here can be used to efficiently and reversibly inhibit the expression of *P. salmonis* genes. This system enables, for example, the knockdown of essential genes, which cannot be knocked out because they are required for bacterial survival, providing insights into their function. Additionally, a multiplex Mobile-CRISPRi platform can be used to silence several genes simultaneously (82). As reported, this approach could be particularly useful for studying bacterial pathogenesis, given that the redundancy of virulence genes can compensate for their individual contributions to a pathogen’s ability to cause disease (83). Thus, Mobile-CRISPRi provides an alternative to traditional engineering approaches and a potentially useful tool to study gene function in this fastidious intracellular bacterium.

## Data Availability

RNA-seq data are available in the Gene Expression Omnibus (GEO) repository with the accession number GSE309378.

## SUPPLEMENTAL MATERIAL

Supplemental figures aem.01560-25-s0001.pdf Figures S1 to S5.

Supplemental methods aem.01560-25-s0002.pdf Additional experimental details.

Supplemental tables aem.01560-25-s0003.pdf Tables S1 to S5.

Table S6 aem.01560-25-s0004.xlsx Genes differentially expressed between IPTG-induced and uninduced sgRNA-*fur1 P. salmonis* strains.

ASM does not own the copyrights to Supplemental Material that may be linked to, or accessed through, an article. The authors have granted ASM a non-exclusive, world-wide license to publish the Supplemental Material files. Please contact the corresponding author directly for reuse.
