# Supplementary material for: Mobile-CRISPRi as a tool for genetic manipulation in the intracellular pathogen Piscirickettsia salmonis
Source: Appl Environ Microbiol. 2025 Dec 22;92(1):e01560-25. doi: 10.1128/aem.01560-25 (PMC12838400; doi:10.1128/aem.01560-25)
Supplement: Supplemental figures — Figures S1 to S5. [file aem.01560-25-s0001.pdf]

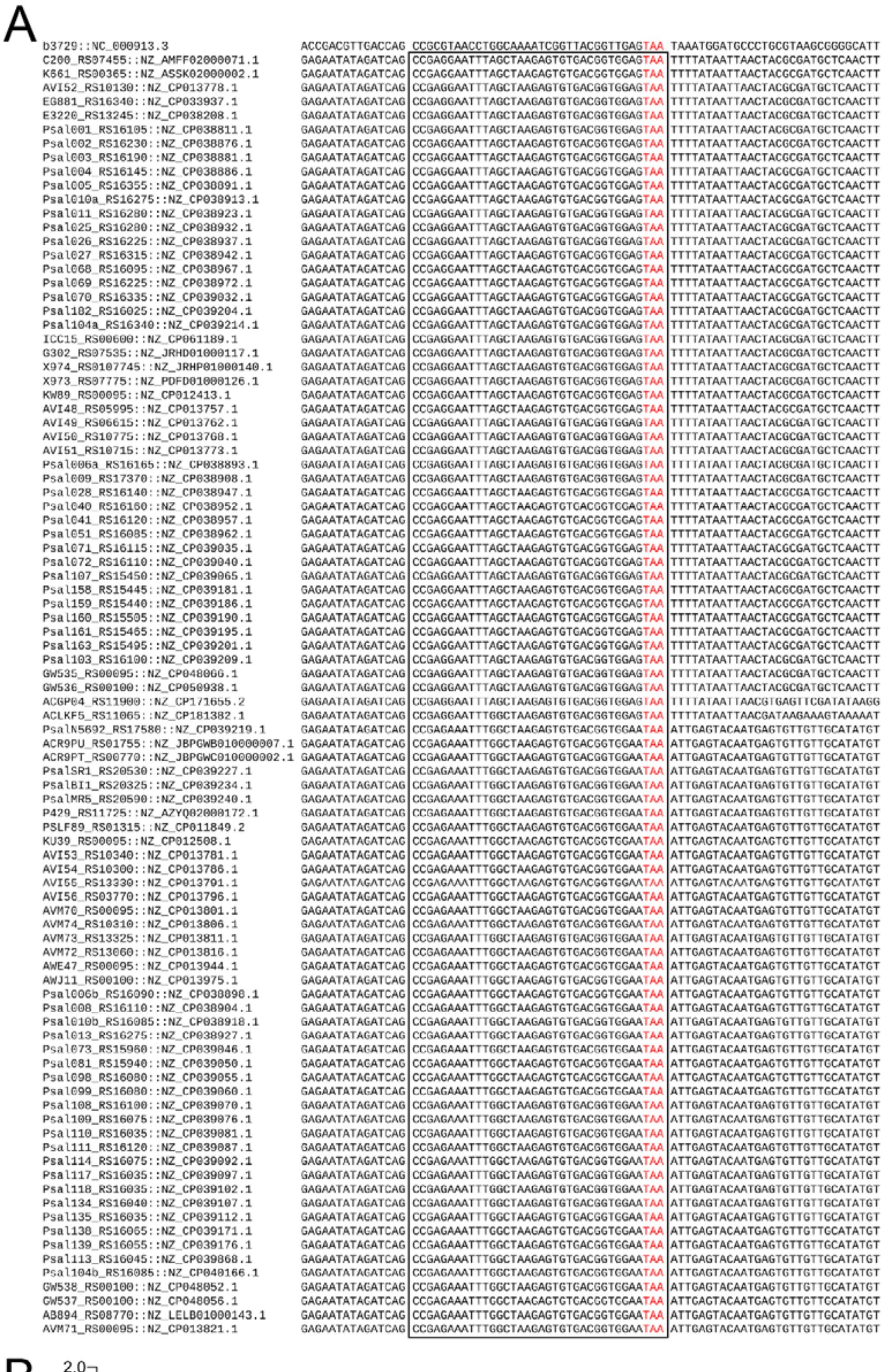
A
B
bits
2.0
1.0
0.0
5
10
15
20
25
30
35
WebLogo 3.7.9

Fig S1. Alignment of *attTn7* box in different *P. salmonis* strains. (A) Alignment of TnsD binding site (*attTn7* box) in 92 available RefSeq genomes of *P. salmonis* (downloaded from the NCBI website https://www.ncbi.nlm.nih.gov/datasets/genome/?taxon=1238, accessed on July 23, 2025). For each genome, the sequence of the *glmS* CDS plus 30 nucleotides downstream was extracted using BEDTools v2.31.1 and aligned. The 36 nucleotide *attTn7* box is marked by a black rectangle and the *glmS* stop codon is shown in red letters. (B) Consensus sequence logo of the *attTn7* box obtained by the alignment of the 92 *P. salmonis* genomes.

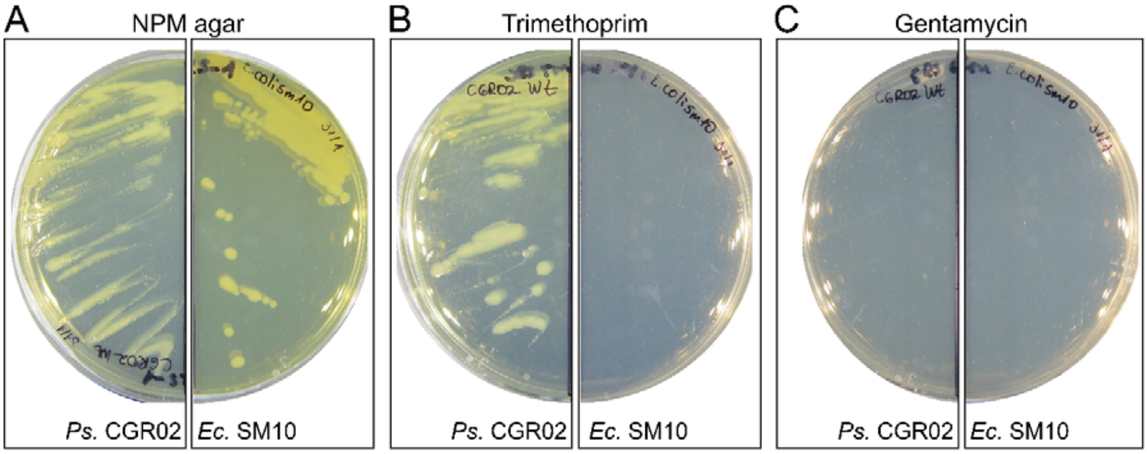

Fig S2. Growth tests for the donor and recipient strains for selection of transconjugants. Culture of *P. salmonis* CGR02 (*Ps.* CGR02) and *E. coli* SM10 (*Ec.* SM10) wild-type strains in the agar medium where the conjugation was conducted (NPM) at 18 ºC (A), the same medium supplemented with trimethoprim (B) and gentamycin (C).

	Max load (DO600)	SD
WT	1.573	0.004
WT IPTG	1.550	0.008
sgRNA-*fur*1	1.519	0.015
sgRNA-*fur*1 IPTG	1.613	0.005
sgRNA-*fur2*	1.442	0.012
sgRNA-*fur2* IPTG	1.526	0.010
	**Generational time (h)**	**SD**
WT	5.664	0.014
WT IPTG	5.385	0.157
sgRNA-*fur*1	6.221	0.469
sgRNA-*fur*1 IPTG	6.464	0.068
sgRNA-*fur2*	4.979	0.108
sgRNA-*fur2* IPTG	5.186	0.052
	**Experimental AUC**	**SD**
WT	90.817	0.541
WT IPTG	91.134	0.249
sgRNA-*fur*1	82.382	0.826
sgRNA-*fur*1 IPTG	84.582	0.515
sgRNA-*fur2*	89.419	0.687
sgRNA-*fur2* IPTG	91.691	0.611

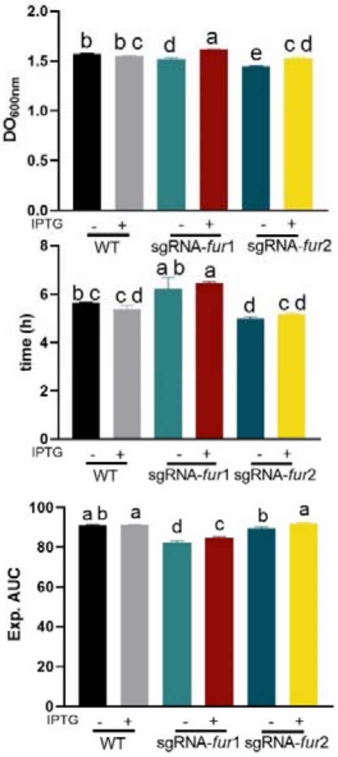

Fig S3. Growth curve parameters of wild-type and *fur*-knockdown strains. Growth curves of *P. salmonis* sgRNA-*fur1*, sgRNA-*fur2* and WT strains with or without IPTG supplementation. The strains were grown in NPB Broth with 400 µM $FeCl_3$. Data was analyzed using the package R Growthcurver to obtain the growth parameters for each individual replicate (n = 3 biological replicates). Letters on the bar graphs indicate statistical differences ($p < 0.05$, evaluated by one-way ANOVA and Tukey multiple comparisons post-test).

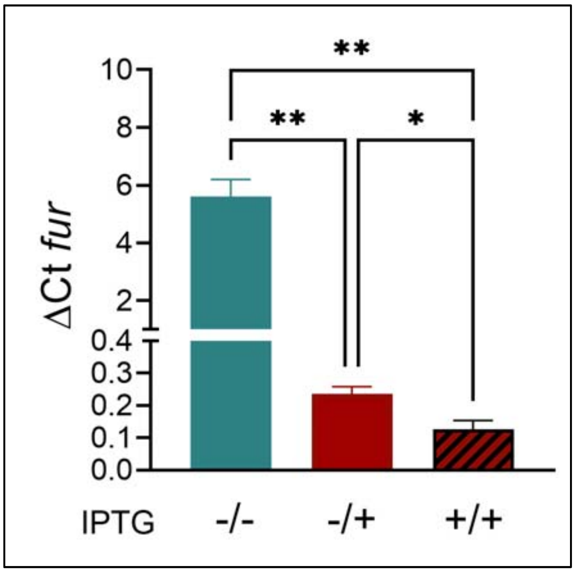

Fig. S4. qPCR quantification of *fur* gene expression in the sgRNA-*fur1* strain after none (-/-), one (-/+) or two (+/+) passages in presence of the inducer IPTG. ΔCt values normalized by the expression of the housekeeping gene *recF* (one-way ANOVA with Tukey's multiple comparisons test, * $p < 0.05$, ** $p < 0.01$, $n = 3$ independent replicates).

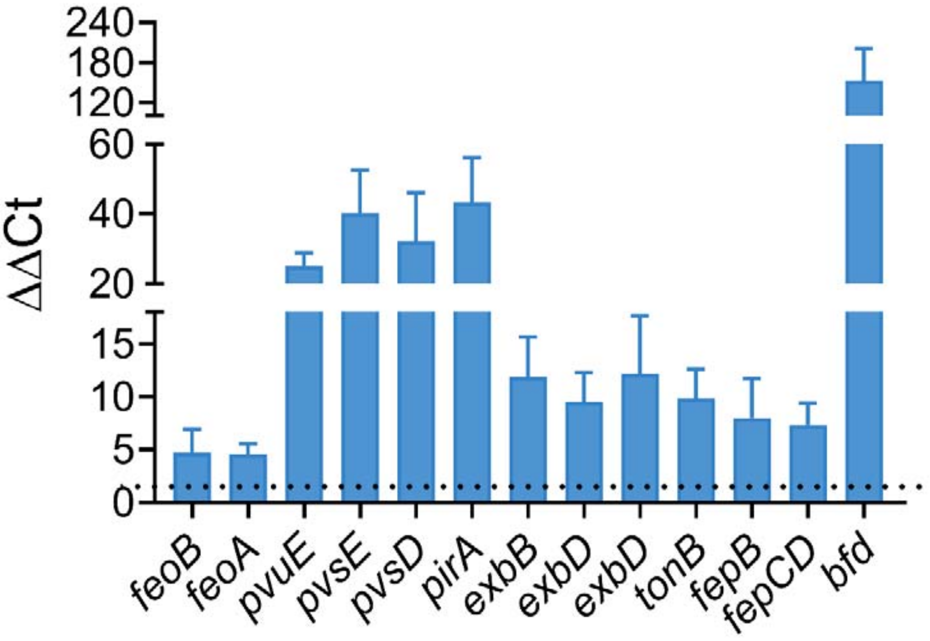

Fig S5. RT-qPCR quantification of transcripts in the sgRNA-fur2 for the upregulated genes identified in the sgRNA-fur1 strain RNA-seq analysis. *P. salmonis* genes are expressed as ΔΔCt values of the sgRNA-fur2 knockdown strain, normalized by the control (non-induced bacteria), and recF as the housekeeping gene. The dotted line indicates a ΔΔCt value of 2.
