# Supplementary material for: Mobile-CRISPRi as a tool for genetic manipulation in the intracellular pathogen Piscirickettsia salmonis
Source: Appl Environ Microbiol. 2025 Dec 22;92(1):e01560-25. doi: 10.1128/aem.01560-25 (PMC12838400; doi:10.1128/aem.01560-25)
Supplement: Supplemental methods — Additional experimental details. [file aem.01560-25-s0002.pdf]

Supplementary methods

1. Transformation of E. coli SM10

pJMP2754, pJMP2774 and pTNS3 plasmid DNA were purified from *E. coli* BW25141 and *E. coli* Pir1 hosts using the EZNA Plasmid Mini Kit II (Omega Bio-tek) following manufacturer's instructions, quantified using Qubit Fluorometric Quantitation System (Life Technologies) and 50 to 75 ng of DNA were used to transform chemically competent *E. coli* SM10 cells following standard protocols. Transformed cells were plated on LB agar plates with gentamicin (30 μg/mL) or ampicillin (100 μg/mL) according to plasmid resistance.

2. Most Probable Number (MPN) assays

For MPN assays, 20 μL of the conjugation mixture per replicate (N = 5 independent matings) was inoculated into 180 μL of NPB supplemented with trimethoprim or with trimethoprim and gentamicin in 96-well plates, and the results were obtained from 12 ten-fold serial dilutions with 3 replicates for each dilution. The plates were then incubated for a period of 8-12 days at 20°C, after which the U.S. Environmental Protection Agency MPN calculator, version 2.0, was used to generate the MPN counts for each sample (1).

3. Growth curve assays

For growth curve assays, stationary state bacteria (OD_{600} = 1.1-1.3) were used as inoculum. 48-well plates were inoculated to a final OD_{600} = 0.01 in 700 μl NPB broth and incubated at 18°C with shaking at 180 rpm. Every growth curve experiment was performed with three technical replicates and 11 biological replicates. Bacterial growth was assessed periodically by measuring the OD_{600} for five days in an Infinite 200 PRO NanoQuant (Tecan, Switzerland).

4. RNA purification

P. salmonis strains sgRNA-fur1 and sgRNA-fur2 were grown for 38 hours (mid-exponential phase) at 18°C in NPB supplemented or not with IPTG 100 μM and then were collected by centrifugation 8000 × g for 10 min at 4°C, supernatants were discarded and the pellets were suspended in 750 μL of TRIzol (Thermo Fisher Scientific) and total RNA extraction was carried out according to manufacturer’s instructions. Four micrograms of RNA were treated

with Turbo DNA-free Kit (Invitrogen) according to standard protocols. Purified RNA was resuspended in 50 µL of nuclease free water and quantified using a Qubit Fluorometric Quantitation System (Life Technologies). RNA quality was assessed using the 2200 TapeStation Bioanalyzer (Agilent Technologies).

5. qPCR assays

PCR conditions were 95°C for 3 min followed by 95°C for 3 s, 62–62.5°C for 10 s and 72°C for 12 s for a total of 35 cycles. Melting curves (0.5°C steps between 65–95°C) ensured that a single product was amplified in each reaction. To determine relative expression levels of genes, the method described by Pfaffl (2) was employed, using gene *recF* as an internal reference gene (housekeeping). At least 3 biological replicates were analyzed, and PCR efficiencies were determined by linear regression analysis performed directly on the sample data using LinRegPCR (3).

6. Protein quantification

To quantify the protein content of the bacteria, 1 mL of the samples was centrifuged at 8000 × g for 10 min at 4°C. The supernatant was then discarded, and the pellet resuspended in PBS containing a protease inhibitor (Sigma-Aldrich). The samples were sonicated in two 30-second pulses, incubating on ice between each pulse, then they were centrifuged again at 10,000 × g for 10 min at 4°C. Quantification was performed using the Qubit Protein Assay Kit (Thermo Fisher), following the supplier's instruction. The concentration of each sample was then determined by interpolation using a calibration curve constructed with 3 internal standards. GraphPad Prism (GraphPad Software, La Jolla, CA, United States) was used for graphical presentation and statistical analysis of data.

References

1. U.S. Environmental Protection Agency. 2013. MOST PROBABLE NUMBER (MPN) CALCULATOR Version 2.0 User and System Installation and Administration Manual.

2. Pfaffl MW. 2001. A new mathematical model for relative quantification in real-time RT–PCR. Nucleic Acids Res 29:e45. doi: 10.1093/nar/29.9.e45.

3. Ramakers C, Ruijter JM, Lekanne Deprez RH, Moorman AFM. 2003. Assumption-free analysis of quantitative real-time polymerase chain reaction (PCR) data. Neurosci Lett 339:62-6. doi: 10.1016/s0304-3940(02)01423-4.
