# Supplementary material for: Mobile-CRISPRi as a tool for genetic manipulation in the intracellular pathogen Piscirickettsia salmonis
Source: Appl Environ Microbiol. 2025 Dec 22;92(1):e01560-25. doi: 10.1128/aem.01560-25 (PMC12838400; doi:10.1128/aem.01560-25)
Supplement: Supplemental tables — Tables S1 to S5. [file aem.01560-25-s0003.pdf]

Table S1. List of primers used in this study.

Primer name	Target	Sequence 5'-3'
27F	Bacterial *16S rRNA*	AGAGTTTGATCCTGGCTCAG
1492R		CGGTTACCTTGTTACGACTT
Tn7R	Transconjugant insert	CACAGCATAACTGGACTGATTTC
glmsps2		GCATGAACATCTTGCACCATT
Fur1sgRNA_F	*fur sgRNA* designed for Mobile-CRISPRi	TAGTTGCGCTCACATGGCGTTGCT
Fur1sgRNA_R		AAACAGCAACGCCATGTGAGCGCA
Fur2sgRNA_F	*fur sgRNA* designed for Mobile-CRISPRi	TAGTAAACATCTTCTGCGCTCACA
Fur2sgRNA_R		AAACTGTGAGCGCAGAAGATGTTT
recF-15880_F	*P. salmonis recF*	CGCCTTCAAGCCAATTGTGG
recF-15880_R		GCAAGCTTTTCACCTTGCCA
dCas9_F	*dcas9* gene	ATTTTGCGGTTGCTTTGCCT
dCas9_R		CCGTCGTTGGAACTGCTTTG
fur-14385_F	*P. salmonis fur* Locus tag AWJ11_14385	CATGTGAGCGCAGAAGATGT
fur-14385_R		ACCACAGCGTGTACAGACCA
feoA-02095_F	*P. salmonis feoA* Locus tag AWJ11_02095	TCTTTACAAGCTGGCACTTCC
feoA-02095_R		GGCGGATACATAGGGCATAA
feoB-02090_F	*P. salmonis feoB* Locus tag AWJ11_02090	ATGCGGCGAACTTAGAAAGA
feoB-02090_R		GATCGGTGCCTTGGTTTTTA
AWJ11_07000_F	*P. salmonis pvsD* Locus tag AWJ11_07000	ACAGTGGTCTTGTGTGGAGT
AWJ11_07000_R		GTGACAGCCCCCATTTGGTA
AWJ11_06980_F	*P. salmonis pvuE* Locus tag AWJ11_06980	AATTGGGCGTTAGACGTCAC
AWJ11_06980_R		GTCATGGAGGACCCAAAGAA
AWJ11_07035_F	*P. salmonis fepB* Locus tag AWJ11_07035	CGGTTAAAGTGCCGGTTAAG
AWJ11_07035_R		CGCCCTACACTTTCAACTCC
exbB-07015_F	*P. salmonis exbB* Locus tag AWJ11_07015	TCAGTTTGTTCGCTGGGGAG
exbB-07015_R		GATGAAGTCGCACCCGATCT
lysA-06985_F	*P. salmonis lysA* Locus tag AWJ11_06985	CCATAATCCGCTTCGAATGT
lysA-06985_R		ACGGTAAAAGGATGGCTGTG
bfd-07380_F	*P. salmonis bfd* Locus tag AWJ11_07380	GAGCTGTGTCGAGGGTATGG
bfd-07380_R		ACACTGTTGACTTAAAGCACCT
AWJ11_07040_F	*P. salmonis fpcCD* Locus tag AWJ11_07040	GCTGCTGCTTTGGTCTTCTG
AWJ11_07040_R		GCCTTGCTGCTCAACATCAC
AWJ11_07010_F	*P. salmonis pirA* Locus tag AWJ11_07010	CGAATGCTTGGTACTTGGCG
AWJ11_07010_R		CTGGCGCACGCAATATCATC
tonB-07030_F	*P. salmonis tonB* Locus tag AWJ11_07030	CCGACAGATTACGGGTGAGG
tonB-07030_R		AGGAAATGGGTTTGCCTGCT
exbD1-07020_F	*P. salmonis exbD* Locus tag AWJ11_07020	ACTCAGTTTGTTCGCTGGGG
exbD1-07020_R		GCTCTGCTTGATGAAGTCGC

exbD2-07025_F	*P. salmonis exbD* Locus tag AWJ11_07025	GCAGACAATCGCAATGGAGAG
exbD2-07025_R		GGTAAATCATCGGAAGCGGC
sodB-09080_F	*P. salmonis sodB* Locus tag AWJ11_09080	AGGTATTGTGGTGCTTGCCA
sodB-09080_R		CTGCCGTATGCGATGAATGC
cydB-08535_F	*P. salmonis cydB* Locus tag AWJ11_08535	AAATAAGCCGCACCATGCAC
cydB-08535_R		ATGGGGGTCGCTTTTGGTAA
acnA-13015_F	*P. salmonis acnA* Locus tag AWJ11_13015	AAGACGCTTGGGTTCGATGT
acnA-13015_R		GCTGGATAAAGCTGTTGCGG

Table S2. List of bacterial strains, their characteristics and usage in this study.

**Strain**	Characteristics and use	Resistance	Reference
E. coli Sm10 *λpir*	(F-) RP4-2-Tc::Mu *recA λpir* Donor strain for conjugation assays	Km	Accession number LMBP 3889. (1)
E. coli Sm10 pTNS3	Donor strain for conjugation assays harboring plasmid with transposition machinery	Km, Amp	This work
E. coli Sm10 pJMP2754	Donor strain for conjugation assays harboring plasmid with "test" Mobile-CRISPRi	Km, Amp, Gm	This work
E. coli Sm10 pJMP2774	Donor strain for conjugation assays harboring plasmid with functional Mobile-CRISPRi	Km, Amp, Gm	This work
E. coli Sm10 pJMP2782:sgRNA-*fur1*	Donor strain for conjugation assays harboring plasmid with functional Mobile-CRISPRi	Km, Amp, Gm	This work
E. coli Sm10 pJMP2782:sgRNA-*fur2*	Donor strain for conjugation assays harboring plasmid with functional Mobile-CRISPRi	Km, Amp, Gm	This work
E.coli Pir1	F- Δ*lac169 rpoS*(Am) *robA1 creC510 hsdR514 endA recA1 uidA(ΔMluI)*::*pir-116.* Plasmid maintenance		Addgene (2)
E. coli BW25141	F-, Δ(*araD-araB*)567, ΔlacZ4787(::rrnB-3), Δ(*phoB-phoR*)580, λ-, galU95, ΔuidA3::pir+, recA1, endA9(del-ins)::FRT, rph-1, Δ(*rhaD-rhaB*)568, hsdR514Plasmid propagation		Addgene (3)
P. salmonis CGR02	WT, genogroup B, receiving strain in conjugation	Tmp	Etecma, Puerto Montt, Chile. (4)
P. salmonis LF-89 (ATCC VR-1361)	WT, reference strain, genogroup B, receiving strain in conjugation	Tmp	(5)
P. salmonis PSCGR01	WT, genogroup B, receiving strain in conjugation	Tmp	Etecma, Puerto Montt, Chile. (4)
P. salmonis NVI5692	WT, genogroup B, receiving strain in conjugation	Tmp	(6)

P. salmonis NVI5892	WT, genogroup B, receiving strain in conjugation	Tmp	(6)
P. salmonis Ps-8079A	WT, genogroup A, receiving strain in conjugation	Tmp	Etecma, Puerto Montt, Chile. (4)
P. salmonis PS12201A	WT, genogroup A, receiving strain in conjugation	Tmp	Etecma, Puerto Montt, Chile. (4)
sfGFP(+)	*P. salmonis* CGR02, transconjugant harboring Tn7 insertion of Mobile-CRISPRi with inducible expression of *dcas9* and constitutive expression of *sfGFP*	Tmp, Gm	This work
sgRNA-s*fGFP*	*P. salmonis* CGR02, transconjugant harboring Tn7 insertion of Mobile-CRISPRi with inducible expression of *dcas9* and gmc6 sgRNA, and constitutive expression of *sfGFP*	Tmp, Gm	This work
sgRNA-*fur1*	*P. salmonis* CGR02, transconjugant harboring Tn7 insertion of Mobile-CRISPRi with inducible expression of *dcas9* and constitutive expression of *fur1* sgRNA	Tmp, GM	This work
sgRNA-*fur2*	*P. salmonis* CGR02, transconjugant harboring Tn7 insertion of Mobile-CRISPRi with inducible expression of *dcas9* and constitutive expression of *fur2* sgRNA	Tmp, Gm	This work
LF-89-sgRNA	*P. salmonis* LF-89, transconjugant harboring Tn7 insertion of Mobile-CRISPRi with inducible expression of *dcas9*, gmc6 sgRNA, and constitutive expression of *sfFGP*	Tmp, Gm	This work
PSCGR01-sgRNA	*P. salmonis* PSCGR01, transconjugant harboring Tn7 insertion of Mobile-CRISPRi with inducible expression of *dcas9*, gmc6 sgRNA, and constitutive expression of *sfFGP*	Tmp, Gm	This work
NVI5692-sgRNA	*P. salmonis* NVI5692, transconjugant harboring Tn7 insertion of Mobile-CRISPRi with inducible expression of	Tmp, Gm	This work

	dcas9, gmc6 sgRNA, and constitutive expression of *sfFGP*		
NVI5892-sgRNA	*P. salmonis* NVI5892, transconjugant harboring Tn7 insertion of Mobile-CRISPRi with inducible expression of *dcas9*, gmc6 sgRNA, and constitutive expression of *sfFGP*	Tmp, Gm	This work
Ps-8079A-sgRNA	*P. salmonis* Ps-8079A, transconjugant harboring Tn7 insertion of Mobile-CRISPRi with inducible expression of *dcas9*, gmc6 sgRNA, and constitutive expression of *sfFGP*	Tmp, Gm	This work
PS12201A-sgRNA	*P. salmonis* PS12201A, transconjugant harboring Tn7 insertion of Mobile-CRISPRi with inducible expression of *dcas9*, gmc6 sgRNA, and constitutive expression of *sfFGP*	Tmp, Gm	This work

Table S3. Plasmids and vectors used in this study.

Plasmid	Detail	Resistance	Reference
pTNS3	Transposition machinery	Amp	H. Schweizer (Addgene # 63127) (2)
pJMP2754	*sfGFP*, *dcas9*, empty sgRNA, GmR	Gm/Amp	J. Peters (Addgene #160666) (7)
pJMP2774	sfGFP, gmc6 sgRNA, *dcas9*, GmR	Gm/Amp	J. Peters (Addgene #160667) (7)
pJMP2782	*dcas9*, empty sgRNA, GmR	Gm/Amp	J. Peters (Addgene # 160668) (7)
pJMP2782:fur1	*dcas9*, *fur1* sgRNA, GmR	Gm/Amp	This study
pJMP2782:fur2	*dcas9*, *fur2* sgRNA, GmR	Gm/Amp	This study

Table S4. Five top-scoring occurrences of the TnsD binding motif in *P. salmonis, E. coli and P mirabilis* genomes.

Motif	Genome ID	Start	Stop	Strand	Score	p-value	q-value	Matched_sequence
***Piscirickettsia salmonis* CGR02**								
NNNNGNAACCTGGCVAARWSBGTNACSGTBGNNNNN	NZ_CP013975.1	19831	19866	+	33.4655	1.24E-11	8.39E-05	CCGAGAAATTTGGCTAAGAGTGTGACGGTGGAATAA
NNNNGNAACCTGGCVAARWSBGTNACSGTBGNNNNN	NZ_CP013975.1	1787346	1787381	-	-6.96552	2.78E-07	0.843	TTTGGTGACTTAGAAAACACGGTGACAGTCGAGCAG
NNNNGNAACCTGGCVAARWSBGTNACSGTBGNNNNN	NZ_CP013975.1	1033790	1033825	-	-10.7069	5.39E-07	0.843	CTATCAAACATTGCCAAGACTGTCACGCTCGTTCAT
NNNNGNAACCTGGCVAARWSBGTNACSGTBGNNNNN	NZ_CP013975.1	132683	132718	+	-11.7241	6.38E-07	0.843	TTGTAAAATTTGGCTAAGTTGGTGACTATTTTATGC
NNNNGNAACCTGGCVAARWSBGTNACSGTBGNNNNN	NZ_CP013975.1	1235994	1236029	+	-13.2931	8.31E-07	0.843	ATGTACAACTTGGCGATGGCGGTGATAGTACGGTTA
***Escherichia coli* K-12**								
NNNNGNAACCTGGCVAARWSBGTNACSGTBGNNNNN	NC_000913.3	3911839	3911874	-	37.9655	4.90E-13	4.50E-06	CCGCGTAACCTGGCAAAATCGGTTACGGTTGAGTAA
NNNNGNAACCTGGCVAARWSBGTNACSGTBGNNNNN	NC_000913.3	3818224	3818259	-	5.37931	2.49E-08	0.114	TTCTGAAATATGGCGAAATCAGTAGCTGTCGCTACC
NNNNGNAACCTGGCVAARWSBGTNACSGTBGNNNNN	NC_000913.3	2669786	2669821	+	1.7931	5.18E-08	0.159	CGCTGCAATTTGGTCAGGACGGTCACGGTAGCGGTT
NNNNGNAACCTGGCVAARWSBGTNACSGTBGNNNNN	NC_000913.3	2687135	2687170	-	-0.27586	7.89E-08	0.16	CGGCGCAAACCGGCAAAATCGGTGACGGTAAAATCT

NNNNGNAACCTG GCVAARWSBGTNA CSGTBGNNNNN	NC_000913.3	1971963	1971998	-	-3.27586	1.39E-07	0.16	GGTGGTAACCTGGCGA ATACCCTGACCATTGAC GGG
***Proteus mirabilis* HI4320**								
NNNNGNAACCTG GCVAARWSBGTNA CSGTBGNNNNN	NC_010554.1	3371818	3E+06	+	32.3103	1.99E-11	0.000163	CCTCGAAACTTGGCTA AATCAGTGACTGTTGA GTAA
NNNNGNAACCTG GCVAARWSBGTNA CSGTBGNNNNN	NC_010554.1	32598	32633	+	11.1552	7.14E-09	0.0293	GGAATGGATTTGGCGA AAACGGTGACAGTGGA ACAA
NNNNGNAACCTG GCVAARWSBGTNA CSGTBGNNNNN	NC_010554.1	3714589	4E+06	-	2.24138	4.71E-08	0.129	ATGAGCAAGTTTGCCA AGGCCGTACCGGTCAT GCAG
NNNNGNAACCTG GCVAARWSBGTNA CSGTBGNNNNN	NC_010554.1	170073	170108	+	-2.58621	1.22E-07	0.25	TTAGGTAATGTGGTTAA AGCAGTGACGGTTGCT GAC
NNNNGNAACCTG GCVAARWSBGTNA CSGTBGNNNNN	NC_010554.1	3457703	3E+06	+	-5.62069	2.15E-07	0.301	AATCATAACCTAGCCA AGGGTGGTAAAGTTGT GCTC

Table S5. Conjugative efficiency of *P. salmonis* strains, shown as the ratio between each sgRNA-*sfGFP* knockdown and its wild-type strain.

Strain	Conjugative efficiency[1]
**Ps-8079A**	2.57×10^{-3}
**PS12201A**	1.00×10^{-1}
**LF-89**	1.00×10^{-7}
**PSCGR01**	3.89×10^{-2}
**CGR02**	$1\text{-}2.57 \times 10^{-1}$

[1]The conjugative efficiency was calculated as the ratio of viable transconjugants grown in NPB supplemented with gentamycin plus trimethoprim (transconjugants), to viable *P. salmonis* cells grown in NPB plus trimethoprim (receptors). The viable number of *P. salmonis* transconjugants was estimated by the Most Probable Number (MPN) method.

References

1. Simon R, Priefer U, Pühler A. 1983. A broad host range mobilization system for in vivo genetic engineering: Transposon mutagenesis in gram negative bacteria. Nat Biotechnol 1:784–791. doi:10.1038/nbt1183-784.

2. Choi KH, Mima T, Casart Y, Rholl D, Kumar A, Beacham IR, Schweizer HP. 2008. Genetic tools for select-agent-compliant manipulation of *Burkholderia pseudomallei.* Appl Environ Microbiol 74:1064-75. doi:10.1128/AEM.02430-07.

3. Datsenko KA, Wanner BL. 2000. One-step inactivation of chromosomal genes in *Escherichia coli* K-12 using PCR products. Proc Natl Acad Sci U S A 97:6640-5. doi: 10.1073/pnas.120163297.

4. Aravena P, Pulgar R, Ortiz-Severín J, Maza F, Gaete A, Martínez S, Serón E, González M, Cambiazo V. 2020. PCR-RFLP detection and genogroup identification of *Piscirickettsia salmonis* in field samples. Pathogens 9:358. doi: 10.3390/pathogens9050358.

5. Fryer JL, Lannan CN, Garces LH, Larenas JJ, Smith PA. 1990. Isolation of a rickettsiales-like organism from diseased coho salmon (*Oncorhynchus kisutch*) in Chile. Fish Pathol 25:107-114. doi:10.3147/jsfp.25.107.

6. Mikalsen J, Skjærvik O, Wiik-Nielsen J, Wasmuth MA, Colquhoun DJ. 2008. Agar culture of *Piscirickettsia salmonis*, a serious pathogen of farmed salmonid and marine fish. FEMS Microbiol Lett 278:43-7. doi: 10.1111/j.1574-6968.2007.00977.x.

7. Banta AB, Ward RD, Tran JS, Bacon EE, Peters JM. 2020. Programmable Gene Knockdown in Diverse Bacteria Using Mobile-CRISPRi. Curr Protoc Microbiol 59:e130. doi: 10.1002/cpmc.130.
